# Cell-Type Specific Determinants of *NRAMP1* Expression in Professional Phagocytes

**DOI:** 10.3390/biology2010233

**Published:** 2013-01-25

**Authors:** Mathieu F. M. Cellier

**Affiliations:** Inrs-Institut Armand-Frappier, 531, Bd des prairies, Laval, QC H7V 1B7, Canada; E-Mail: mathieu.cellier@iaf.inrs.ca; Tel.: +1-450-687-5010 (ext. 4681); Fax: +1-450-686-5301

**Keywords:** Natural resistance-associated macrophage protein (Nramp), SLC11A1, regulation of gene expression, myeloid differentiation, genetic variation, HL-60 cells, C/EBPβ, PU.1

## Abstract

The Natural resistance-associated macrophage protein 1 (Nramp1 or Solute carrier 11 member 1, Slc11a1) transports divalent metals across the membrane of late endosomes and lysosomes in professional phagocytes. Nramp1 represents an ancient eukaryotic cell-autonomous defense whereas the gene duplication that yielded *Nramp1* and *Nramp2* predated the origin of Sarcopterygians (lobe-finned fishes and tetrapods). *SLC11A1* genetic polymorphisms associated with human resistance to tuberculosis consist of potential regulatory variants. Herein, current knowledge of the regulation of *SLC11A1* gene expression is reviewed and comprehensive analysis of ENCODE data available for hematopoietic cell-types suggests a hypothesis for the regulation of *SLC11A1* expression during myeloid development and phagocyte functional polarization. *SLC11A1* is part of a 34.6 kb CTCF-insulated locus scattered with predicted regulatory elements: a 3' enhancer, a large 5' enhancer domain and four elements spread around the transcription start site (TSS), including several C/EBP and PU.1 sites. *SLC11A1* locus ends appear mobilized by ETS-related factors early during myelopoiesis; activation of both 5' and 3' enhancers in myelo-monocytic cells correlate with transcription factor binding at the TSS. Characterizing the corresponding *cis*/*trans* determinants functionally will establish the mechanisms involved and possibly reveal genetic variation that impacts susceptibility to infectious or immune diseases.

## 1. Introduction

Nramp was identified by positional cloning of the dominant gene responsible for the macrophage quantitative trait of natural resistance to infection by unrelated bacterial and protozoan pathogens [1].

Nramp proteins are classified as Solute carriers 11 (Slc11) that mediate chemiosmotic uptake of divalent metals such as ferrous iron (Fe^2+^) and manganese (Mn^2+^) [2]. The Nramp family comprises proteins displaying 30% amino acid sequence identity over a hydrophobic core that spans 10 transmembrane segments (TMS) [3]. Nramp transporters catalyze proton-dependent divalent metal import into the cell cytoplasm either from the cell surface or a subcellular compartment. Remote ancestry of the Nramp family has been traced by sequence analyses to a superfamily of structurally conserved but otherwise diverse families of cation-dependent membrane transporters with inverted topological symmetry [4]. Accordingly, in homology threading three-dimensional models, Nramp-specific residues directly involved in proton-dependent divalent metal import occupy pseudo-symmetric positions at the predicted binding sites for cations. Also, structural studies of synthetic peptides corresponding to the pseudo-symmetric TMS1 and TMS6 showed they behave not like typical TMS but rather that they exhibit hinge-like elements in their middle, corresponding to Nramp-specific residues required for proton-dependent divalent metal import [5]. Hence, the structural origin of Nramp is very ancient, and the Nramp family is widespread among prokaryotes.

Bacterial Nramp homologs function as proton-dependent Mn transporters (MntH) but display polyphyletic origins that suggest derived evolution. One group of MntH sequence is restricted to anaerobic micro-organisms; another group possibly emerged later is widespread among Bacteria and includes Archaea, whereas the third group was apparently derived by horizontal gene transfer from a eukaryotic source and is more prevalent among bacteria associated with eukaryotic cells [6]. Few eukaryotic organisms possess a pair of *Nramp* genes such as the amoeba *Dictyostelium discoideum*, the read alga *Cyanidioschyzon merolae* and the lower plant (moss) *Physcomitrella patens* and fungi such as *Chytridiomycota*, *Kickxellomycotina* and *Mucoromycotina* [7] (DOE JGI Mycocosm). These parologous genes whose origin predates the divergence of animals, plants and amoebae were named prototype and archetype Nramp. Prototype Nramp are mainly found in unicellular eukaryotes and yeast (e.g., *Saccharomyces cerevisiae*) and include the likely source of gene transfer towards prokaryotes. Archetype Nramp are found in multi-cellular organisms and yielded in animals Nramp1 and Nramp2. In the amoeba *D. discoideum* which grazes on bacteria, both prototype and archetype Nramp contribute to cytoplasmic iron uptake and host defense against bacterial invasion [8].

*D. discoideum* archetype Nramp (DdNr1) is expressed in intracellular vesicles of the endo-lysosomal pathway. DdNr1 is recruited to phagosomes and macropynosomes where it mediates resistance to invasion by diverse intracellular pathogens such as the Gram positive and negative species *Mycobacterium* and *Legionella*, respectively. DdNr1 transport of divalent metals such as ferrous iron out of the phagosome towards the cytoplasm is supported by the electrogenic V-H^+^ ATPase [9]. The intracellular location and activity of *D. discoideum* archetype Nramp (DdNr1) are thus highly similar to animal Nramp1 [10].

In contrast, *D. discoideum* prototype Nramp (DdNr2) is expressed in the membrane of the contractile vacuole [8]. Deletion of each of *DdNr1,2* affects the growth of *D. discoideum* in conditions of iron depletion and/or overload. Both proteins co-localize with the electrogenic V-H^+^ ATPase that extrudes protons from the cytoplasm, so that H^+^ can re-enter as a driving force for metal uptake. Food starvation induces a developmental process in which the amoeba recycles intracellular material to differentiate and produce resistant spores. *D. discoideum* development is perturbed as a result of deletion of either prototype or archetype *Nramp* genes [8]. The data imply non redundant functions for *D. discoideum* Nramps, and suggest that the ancestral eukaryote gene duplication enabled diversification from nutritive function (prototype) to nutriprive activity (archetype). In fact, archetype Nramp exerts both functions, for phagocytic meal and resistance to environmental conditions or intracellular infection. Such dual role is very similar to Nramp1 roles in recycling iron from ingested erythrocytes and depriving ingested microbes from direct access to Fe and Mn inside the phagosome.

## 2. The Marine Origins of Nramp1

Nramp1 (Slc11a1) was characterized as a divalent metal importer expressed specifically in the membrane of late endosomes/lysosomes of professional phagocytes [11]. It is parologous to the Divalent metal transporter 1 (Dmt1, aka Nramp2 or Slc11a2) which is expressed ubiquitously and in membranes at the cell surface or in recycling endosomes. Both proteins are Slc11 carriers catalyzing proton-dependent uptake of divalent metals including Fe^2+^ and Mn^2+^ [2]. Nramp2/Dmt1 is essential for animal survival [12] and mediates intestinal iron absorption taking advantage of gastric acidification in conjunction with the activity of the duodenal cytochrome b which reduces iron to the ferrous divalent form [13,14]. Nramp2 is required for iron metabolism and erythropoiesis [12]. Human *Nramp2/Dmt1* mutations are responsible for microcytic anemia with hepatic overload in humans resulting from Transferrin cycle dependent defect leading to iron accumulation within endosomes [15].

**Figure 1:**
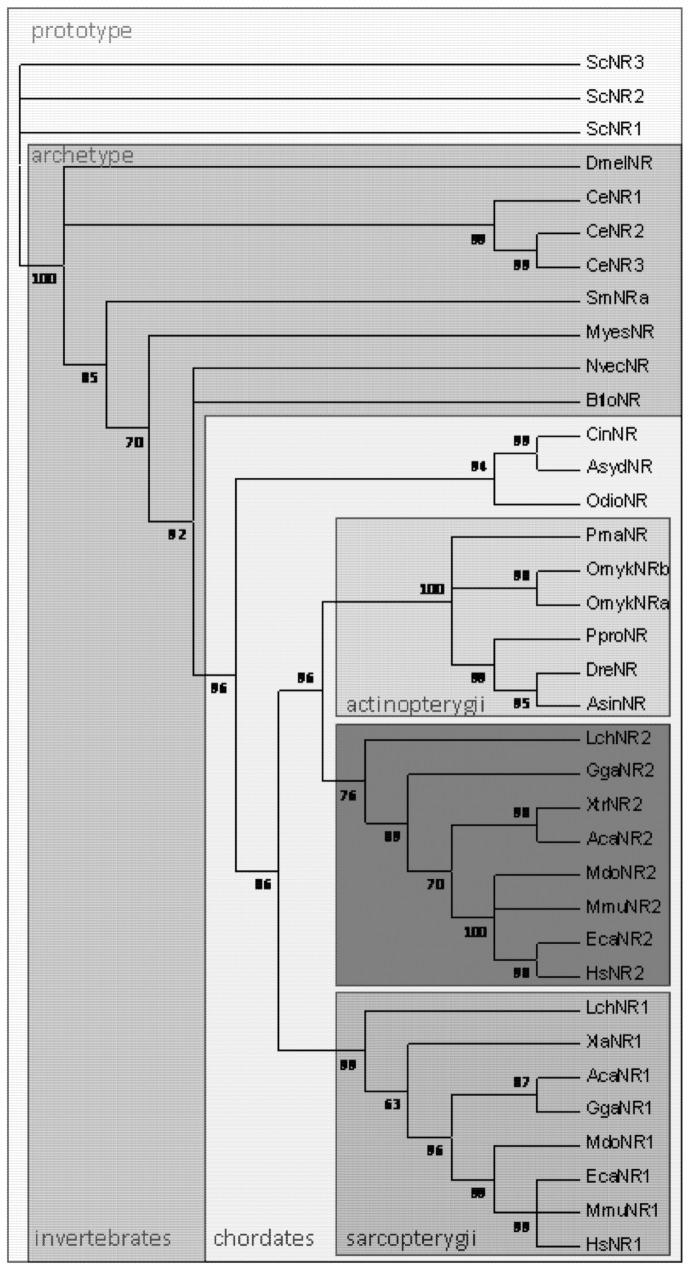
Phylogenetic distribution of *Nramp* in eukaryotes (Opisthokonta). Multicellular organisms possess archetype Nramp whereas *Saccharomyces cerevisiae* retained only prototype Nramp. In chordates, Nramp1 & Nramp2 parologs are present in Sarcopterygians, including lobe-finned fish and tetrapods. Clustering of Sarcopterygian Nramp2 and Actinopterygian Nramp implies *Nramp* duplication occurred before the divergence of Sarcopterygians (adapted from [7]). Prototype Nramp: Sce *Saccharomyces cerevisiae*; Archetype Nramp: Invertebrates Dme, *Drosophila melanogaster*, Ce *Caenorabditis elegans*, Sm *Schistosoma mansoni*, Myes *Mizuhopecten yessoensis*, Nvec *Nematostella vectensis*, Bflo *Branchiostoma floridae*; Chordates Cin *Ciona intestinalis*, Asyd *Ascidia sydneiensis*, Odio *Oikopleura dioica*; Actinopterygii Pma *Pagrus major*, Omyk *Oncorhynchus mykiss*, Ppro *Pimephales promelas*, Dre *Danio rerio*, Asin *Acipenser sinensis*; Sarcopterygii Lch *Latimeria chalumnae*, Xla *Xenopus laevis*, Aca *Anolis carolinensis*, Gga *Gallus gallus*, Mdo *Monodelphis domestica*, Mmu *Mus musculus*, Eca *Equus caballus*, Hsa *Homo sapiens*.

The gene duplication that yielded *Nramp1* and *Nramp2* can be traced to the origin of Sarcopterygians. Genome sequencing of the lobe-finned fish *Latimeria chalumnae* (Coeloacanth, Broad Institute) revealed the coding of Nramp1 and 2 parologs similar to those founds in tetrapods, whereas Actinopterygian (ray-finned) fishes all possess only (one or several copies of) Dmt1/Nramp2 homolog(s) (Figure 1). Animal Nramp proteins contain 12 predicted transmembrane segments (TMS) organized in two domains, repeated 5TMS protomers which are topologically inverted and form the conserved hydrophobic core known as LeuT-fold, plus two C-terminal TMS [16]. *L. chalumnae* Nramp1 protein sequence displays more than 71% amino acid identity with tetrapod Nramp1 orthologs (frogs, lizards, birds and mammals), and up to 70% amino acid identity with Nramp2/Dmt1 parologs. In contrast, *L. chalumnae* Nramp2 protein sequence displays about 80% identity with both tetrapod and ray-finned fish orthologs. Five sites in the predicted TMS2, 3, 6, 8 and 10 show specific variations whose co-occurence distinguishes Nramp1 orthologs from Nramp2/Dmt1 parologs (not shown).

The coelacanth diverged early from the Sarcopterygian lineage (most ancient specimens found ca 420 million years ago, Mya) [17]. From a phylogenetic point of view lungfishes and coelacanths form a monophyletic sister-group that comprises the closest living relative of tetrapods [18]. Sarcopterygians differ from all other fish by their fins, which are borne on fleshy, lobelike, scaly stalks extending from the body (lobe-finned fish). Their pectoral and pelvic fins have articulations resembling those of tetrapod limbs, including humerus and femur, which evolved into arms and legs of the first tetrapod land vertebrates, amphibians. Notably, all Sarcopterygians possess teeth covered with true enamel [17].

Iron deposition into mature enamel is important for tooth constitution and Fe is the only element detected as deposited by ameloblasts outlining the enamel surface of the teeth [19]. Existing data indicate that in mice, covering teeth with true enamel requires iron transport from blood vessels to ameloblasts and that co-regulation of the iron importer Nramp2 (Dmt1, Slc11a2) and the iron exporter Ferroportin-1 (Fpn1, Slc40a1) can contribute to the Fe deposition process [19]. This process is regulated by the nuclear factor erythroid-2-related factor 2 (Nrf2) which coordinates a broad range of cytoprotective transcriptional responses to oxidative and electrophilic stresses [20] as well as iron efflux from macrophages [21]. Nrf2 is also required for hematopoietic stem progenitor cell (HSPC) survival and for myeloid development [22,23,24]; it is one of the regulators most frequently represented in macrophage-related gene signatures [25].

In bony fishes, Fe concentrations in the enameloid were related to the phylogeny of fish and it was proposed that the mechanism of Fe concentration appeared early and disappeared later in the course of fish evolution [26]. Regarding the time of emergence of the parologs Nramp1 and 2, ray-finned fish Nramp homologs segregate clearly with tetrapod Nramp2 [7]. Four of the five sites whose co-variation distinguishes tetrapod Nramp1 from Nramp 2 show residues that are identical in fish Nramp and Nramp2 (25/25 sequences); thus the possibility that Slc11 gene duplication dates back to the so-called “two-round whole genome duplications” implicated at the base of all extant vertebrates [27], with maintenance of both parologs only in Sarcopterygii, remains preferred. Alternatively, occurrence of *Nramp* gene duplication later at the time of emergence of Sarcopterygii would imply highly accelerated sequence evolution for Nramp1.

It might thus be suggested that ensuring novel house-keeping functions such as iron deposition in dental enamel contributed to maintaining both *Nramp1* and *2* genes in Sarcopterygii. Expression of Nramp2 in the apical membrane of ameloblasts combined to Fpn1 localization in the basolateral membrane would provide a setting that resembles intestinal enterocytes, allowing trans-cellular movement of iron and leading to dental deposition of the metal. It should be instructive to further study the tissue specificity of lobe-finned fish *Nramp1* and *2* gene expression as well as and their relative contribution to host defense against infections.

## 3. *SLC11A1* Genetic Factor of Resistance to Tuberculosis

The professional phagocyte phenotype of natural resistance to infection by intracellular pathogens which is shared by mouse macrophages [1] and the amoeba *D. discoideum* [9] implies that Nramp (Slc11) function in host defense predates the gene duplication that produced Nramp1 and 2. However, the transition from marine to terrestrial environment and morphological development of tetrapods may have selected an efficient function to prevent infections, including by novel aerial microbes [7]. Accordingly, evidence for pathogen-driven selection of alleles conferring resistance to infection might exist in current populations.

*M. tuberculosis* (Mtb) is a successful pathogen, responsible for the worldwide human infection tuberculosis (TB), which is latent in more than 2 billion people. Up to 10% may develop TB during their lifetime; each year untreated infections cause the death of over 1.5 million people [28,29]. Mtb is an obligate human intracellular pathogen that transmits efficiently through aerosol. Unless innate genetic susceptibility, primo-infection stimulates both innate and adaptive immunity which mount a potent antimicrobial and immunogenic response that blocks pathogen progression and reaches a state of equilibrium, where intracellular bacteria cannot grow and may enter dormancy. Reactivation of the pathogen occurs when immunological equilibrium is lost, and may either be contained again by host or lead to cavitary TB, inflammation, high bacterial load and transmission; so far it is unknown how to achieve sterile eradication of bacteria from the infected host [28,29,30].

### 3.1. Host-Pathogen Co-Adaptation

In general, immunity to mycobacterial species depends on the activation of macrophage microbicidal activities through the IL-12/IL-23–IFN-γ axis. Genetic deficiencies in *IL-12Rβ1*, *IFN-γR1*, *IL-12p40*, *STAT-1*, *IFN-γR2* or *NEMO* were traced back to a rare congenital syndrome, Mendelian susceptibility to mycobacterial diseases involving weakly virulent species [31] or to disseminated TB (*IL-12Rβ1*) [32]. Ingestion of Mtb by pulmonary macrophages and dendritic cells induces proinflammatory cytokines secretion, including interleukin (IL)-12, IL-1β, and Tumor necrosis factor (TNFα). IL-12 stimulates a T-cell helper 1 (T_H_1) response that in turn promotes M1 macrophage microbicidal activities; in addition TNFα broadly modulates macrophage activities such as cytokine and chemokine secretions, microbial killing, programmed necrosis or apoptosis. Despite this antimicrobial potential anti-inflammatory cytokines including IL-10 and TGF-β can also be produced by Mtb-infected macrophages, translating an alternative (or M2) state of activation. The resulting downregulation of proinflammatory cytokines and T-cell proliferation and activation leads to a balanced response, which contains bacteria unless external factors perturb that equilibrium and reactivate infectious disease [30,33].

### 3.2. SLC11A1 Candidate Functional Polymorphisms

Strong evidence supports a critical role for genetic factors in susceptibility or resistance to TB but the mechanisms involved remain elusive. Host resistance to TB is a complex trait due to an intricate balance of host-Mtb interactions involving multiple genes and compounding factors (e.g., phenotype definition, study design, human and microbial population genetic heterogeneity and linkage disequilibria, socio-economical determinants, environmental factors such as nutrition, co-infections, epigenetics, ...). TB resistance has been associated with several gene polymorphisms but with low consistency between replicate studies, while most clinical association results are not yet validated functionally by molecular studies [34,35,36].

*NRAMP1* may be a factor of critical importance for host defense to the development of TB because several *NRAMP1*/*SLC11A1* genetic polymorphisms were consistently associated with TB resistance/susceptibility in many studies, including the disseminated form of pediatric TB [37,38,39]. *SLC11A1* consistent association with host resistance to developing TB however has not established whether this link regards progression from latent infection to active TB disease or susceptibility to primo-infection [34,37,38,40].

Genetic variants under evolutionary selection often have accumulated to relatively high frequencies, which might represent an equilibrium between benefits procured for TB resistance and high risk of other diseases, such as adverse consequences of microbicidal activities [36]. Significant associations but moderate in strength were established with pulmonary TB in different populations, most consistently in Asian populations, also in African and western populations and regarding strains from distinct lineages of Mtb [37,38].

Polymorphisms at the 3' end of *SLC11A1* (D543N, 1729 + 55del4) show stronger associations for western populations, and the frequency of the non coding deletion allele 1729 + 55del4 [41] correlates positively with ancient urbanization [42]. This suggested that longer time period of contact between humans and Mtb favoured selection of resistance alleles. As increased human density could favor aerosol transmission and the evolution of more virulent Mtb strains, such pathogen pressure could drive the increase in frequency of *SLC11A1* resistant alleles by natural selection [35,42,43]. Although the functional basis for selecting such allele remains unknown it is worth noting that D543N, 1729 + 55del4 polymorphisms lie in a region of *SLC11A1* that is predicted to contain regulatory elements (Section 5.2.1).

In few instances *SLC11A1* genetic polymorphism was correlated functionally in macrophages. Hence, the polymorphism -274C/T is a silent nucleotide substitution in codon 66 (Phe) of *SLC11A1* exon 3, which was identified as a major risk factor to acquire pediatric TB [39]. To study the possible impact of the *SLC11A1* -274C allele on protein function, an assay measuring intracellular recruitment to the phagosome of the macrophage late endosomal/lysosomal marker mannose 6-phosphate receptor (M6PR) was employed [44]. Mouse macrophages expressing a functional Nramp1 control the intracellular fate of diverse pathogens such *M. bovis* BCG, *Salmonella enterica* Serovar Typhimurium and *Leishmania donovani* albeit in different ways, depending on the pathogen intracellular strategy [10].

Soon after invading macrophages *Salmonella* establishes a replicative niche by subverting the normal process of phagolysosomal maturation and excluding for instance, the M6PR from the enclosing vacuole membrane. Assaying M6PR recruitment to the phagosome in human macrophages provided readout of SLC11A1 activity suggesting a link between *SLC11A1* -274C allele and decreased innate resistance to pediatric TB [45]. One possibility may be that this synonymous polymorphism impacts translational efficiency [46]; also, prediction of a regulatory element located 150 bp upstream of the polymorphism -274C/T lying in a domain of open chromatin, epigenetically marked in CD14^+^ monocytes (MNs) and monocyte-derived macrophages (MDMs), might suggest interference with gene regulation (Section 5.2.3).

Hence, several *SLC11A1* polymorphisms associated with TB represent potential regulatory variants rather than non-synonymous SNPs altering protein activity. Among other effects, the impact of genetic variation on chromatin structure and the transcriptional machinery is of significance. The combinatorial nature of gene promoters and chromatin modifiers implies that non coding polymorphisms may be active only in a target cell (e.g., macrophage) or a given immune context (pro- or anti-inflammatory). In both human histiocytic U-937 cells and the monocytic cell line THP-1 *SLC11A1* variant -237C stimulates promoter activation, while hypoxia-inducible factor 1 alpha (HIF-1α) regulates *NRAMP1* expression levels by interacting with a microsatellite repeat under evolutionary selection pressure in the promoter region [47,48,49].

The *SLC11A1* promoter region contains several polymorphisms influencing gene expression. The role of a (GT)_n_ microsatellite repeat consisting of 9 identified allelic variants was characterized in details regarding the two most frequent alleles. These alleles differ by one GT repeat, a variation that affects *SLC11A1* promoter activity: allele 3 contains 9 repeats; it is the most frequent allele and drives higher gene expression levels in transient transfection assays using U-937 and THP-1 cells, while allele 2 with 10 repeats has higher Z-DNA-forming propensity but drives lower expression levels in transient transfection assays [47,48,49,50].

Z-DNA formation can facilitate transcriptional initiation and activation. This left-handed conformation of the double helix may be formed at TG repeats within promoters upon remodeling of a mononucleosome by the human SWI/SNF complex [51]. Interestingly in THP-1 cells, *SLC11A1* allele 3 but not allele 2 bound the transcription factors ATF-3 and c-Jun [50]. *SLC11A1* allele 3 transcriptional activation was decreased by co-transfecting *ATF-3*, an effect that was amplified in cells activated with bacterial lipopolysaccharide (LPS) suggesting that ATF-3 dimer may act as repressor [50] (Section 4.4).

Alleles other than allele 3 were significantly associated with an increased risk of TB, independent of disease type, with a meta-analysis mean odd ratio of 1.31 comparing cases to controls (95% confidence interval: 1.59–1.08) [37]. Impaired *SLC11A1* expression may contribute to limit macrophage pro-inflammatory potential, and consequently, reduce formation of nitric oxide (NO), tumour necrosis factor-alpha and interleukin-6 and increasing interleukin-10 production [52]. It has previously been shown in mice that lack of Nramp1 limits macrophage lipocalin expression [53], production of NO [54,55] and intracellular signaling [56,57]; it also affects tissue Fe distribution and the expression of liver genes involved in Fe metabolism [58]. Conversely, absence of Nramp1 was associated with enhanced production and signaling by the anti-inflammatory cytokine IL-10 [59] and *SLC11A1* genetic polymorphism was correlated with variation in LPS-induced IL-10 secretion [60]. Because Fe metabolism is central to macrophage function, and given the impact of cytoplasmic Fe on ROS-based signaling, it is possible that NRAMP1 cell-autonomous activity at the phagosomal membrane affects macrophage inflammatory potential, hence exerting pleiotropic effects on immune response [61,62,63,64,65]. It remains to be established with statistical significance whether the relatively high frequencies of *SLC11A1* promoter (GT)_n_ repeat alleles 2 and 3 represent an equilibrium between the survival advantage of TB resistance and increased risk of auto-immune and inflammatory diseases such as type 1 diabetes [36,66].

### 3.3. Up-Regulation of SLC11A1 Expression by Hypoxia-Induced Factor 1α

The hypoxia-inducible factor alpha (HIF-1α) represents a strong candidate transcriptional activator binding at *SLC11A1* promoter (GT)_n_ repeat. HIFs are O_2_ sensitive transcription factors mediating transcriptional adaptation to hypoxic environments [67]. When stabilized under low O_2_, HIFs can translocate to the nucleus and dimerize with their obligate partner Aryl hydrocarbon receptor nuclear translocator (ARNT) a.k.a. HIF-1β , then recruit coactivators such as histone acetyltransferase (HAT) activities (e.g., CBP/p300). HIF heterodimers activate genes involved in adaptation to hypoxic stress through recognition of and binding to hypoxia-response elements in their promoter. More broadly, HIFs regulation senses oxygen availability, redox status, nutrient availability, as well as inflammatory signals. Hence HIFs integrate metabolic and innate immune responses to infection and inflammation; HIF1α activity predominates in M1 macrophages whereas HIF-2α is preponderant in M2 macrophages [67].

HIF-1α is key for M1 microbicidal macrophage functions since HIF-1α basal activity can be stimulated in non hypoxic conditions by a variety of compounds such as growth factors, cytokines, nitric oxide, LPS and a range of infectious microorganisms [68,69]. NF-ΚB activity is required for *HIF-1α* gene expression and for HIF-1α protein accumulation in response to hypoxia; also, HIF-1α is activated during *in vitro* differentiation of MNs into macrophages. Microbial pathogens such as Gram-positive (Group A *Streptococcus*, *Staphylococcus aureus*) and Gram-negative (*Pseudomonas aeruginosa* and *Salmonella* Typhimurium) bacteria, as well as protozoan parasites (*Toxoplasma gondii*, *Leishmania amazonesis*) and viruses induce HIFs in normoxia [68,69].

*SLC11A1* proximal promoter (GT)_n_ repeat variation regulates allele expression. This microsatellite contains two predicted hypoxia responsive elements and demonstrates Z-DNA-forming ability both *in vitro* and *in vivo*. Transient co-transfections in Baby hamster kidney cells revealed that under normoxic conditions HIF-1α induced up to 4-fold increase in luciferase expression while HIF-2α could not transactivate *NRAMP1* promoter, and *SLC11A1* expression levels after transfecting Chinese hamster ovary cells depended on the presence of HIF-1α. In murine macrophages co-transfected with HIF-1α and *SLC11A1* proximal promoter transcriptional activity was upregulated in response to hypoxia mimetics and HIF stabilizers (CoCl_2_, iron chelator dipyridyl, nitric oxide). The microsatellite allele 3 binds directly the transcriptional regulator HIF-1α/ARNT heterodimers *in vitro*, and *in vivo* when THP-1 macrophages are activated by pathogen or proinflammatory signals [49].

Current genetic data thus suggest that *SLC11A1* promoter *cis-*acting regulatory variation as well as synonymous coding polymorphism can possibly contribute to heritable differences in gene/protein activity that may account for increased resistance/susceptibility to TB. In this regard, appraisal of all the regions involved in the transcriptional regulation of *SLC11A1* will facilitate localizing functional SNPs that may influence with gene expression.

## 4. *SLC11A1* Proximal Promoter Controls Myelo-Monocytic Expression

A hallmark of *SLC11A1* (*NRAMP1*) is tissue-specific expression, limited to the myelo-monocytic lineage [1,70]. The human gene and protein were found expressed essentially in polymorphonuclear neutrophils, MNs, macrophages [70,71,72]. Hence, NRAMP1 function is devoted like its murine ortholog to mediate divalent metal import into the cytoplasm of professional phagocytes, acting in the membrane of phagosomes that may contain recycled erythrocytes [73,74] or infectious microbes [11,75]. Based on the murine model *SLC11A1* expression is thus likely regulated by factors involved in myeloid cell differentiation and/or which may affect phagocyte polarization toward pro- or anti-inflammatory phenotypes [25].

### 4.1. Expression in Mature Mononuclear and Polynuclear Phagocytes

**Figure 2:**
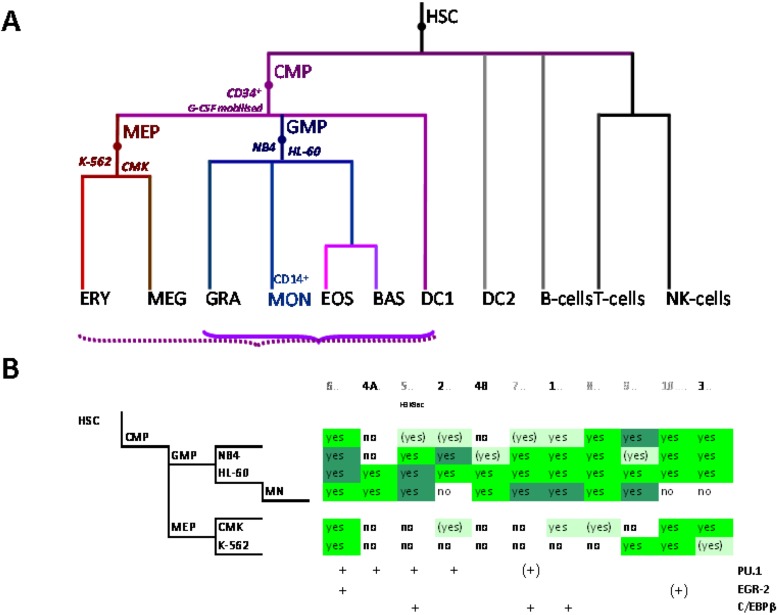
Control of *SLC11A1* gene expression during myelopoiesis. (**A**) Simplified hematopoietic scheme including mature cell types and highlighting the committed progenitors of the myeloid pathway: common myeloid progenitor (CMP), megakaryo-erythrocytic progenitor (MEP) and granulocyte-macrophage progenitor (GMP) as well as the cell-types whose chromatin state was examined as a developmental proxy, respectively G-CSF mobilized CD34^+^ cells, K-562 & CMK and HL-60 & NB4. ERY, erythrocytes, MEG megakaryocytes, GRA granulocytes, (CD14^+^) MON monocytes, EOS eosinophils , BAS basophils, DC1 dendritic cells 1, DC2 dendritic cells 2, B-Cells B lymphocytes, T-cells T lymphocytes, NK-cells natural killer cells (adapted from [76,77]). Parentheses indicate myeloid lineages in which *NRAMP1* locus may be competent for transcription (dotted) or transcriptionally active (plain). (**B**) Summary among myeloid cell-types of 11 ENCODE DNAse I hypersensitive sites showing myelo-monocytic selectivity (except #5, marked by H3K9ac, Figure 4D): #1–4, myelo-monocytic specific, #6 myelo-monocytic enriched, #7–10 strong signal in myelo-monocytic cells. Color-coding for sensitivity to DNAse I, darker for more intense signal. Also indicated, the DNAse I hypersensitive areas that comprise binding sites for the transcription factors PU.1, C/EBPβ or EGR-2 in monocyte-derived macrophages [80].

The myeloid lineage is part of the haematopoietic system which comprises blood cells and is organized hierarchically. Under the current paradigm of hematopoietic differentiation blood cell types arise from hematopoietic stem cells (HSC) which can self-renew or evolve through multiple progenitor and intermediate maturation states into 12 terminally differentiated cell types [76,77]. Phenotypically, distinct lymphoid and myeloid branch points from the HSC occur early during blood cell development, albeit probably not simply dichotomously [78]. Progeny of the common lymphoid precursor (CLP) include B cells, T cells, NK cells and plasmacytoid dendritic cells (DCs) whereas the megakaryocyte-erythroid progenitors (MEPs) and granulocyte-macrophage and myeloid DC (mDC) progenitors (GMP-DC) are generated from common myeloid precursors (CMPs, Figure 2A) [76,79].

mRNA profiling of intermediate and terminally differentiated cell types revealed that genes are tightly coexpressed in modules which are restricted to specific lineages or common to multiple hematopoietic lineages and interconnected. Five dominant phenotypes: HSPCs, differentiated erythroid cells, granulocytes/MNs, B cells and T cells are distinguished by a set of differentially expressed genes specific to each lineage as compared to the others [76]. Analyses in HSPCs showed that the promoter of genes expressed at high level in mature granulocytes and MNs may be bound early in hematopoiesis by factors that specify and maintain differentiation, such as PU.1 and the CAAT enhancer binding protein (C/EBP) β [76].

*SLC11A1* high levels of expression in blood neutrophils and MNs prompted testing the promyelocytic cell line HL-60 as a model to study *SLC11A1* regulation during either monocytic or granulocytic differentiation pathways using various pharmacological agents [70]. *SLC11A1* transcript accumulation was detected co-induced with selected marker genes after 4–6 days differentiation into macrophage- (Phorbol miristate acetate, PMA, *MCSF-R*), monocyte- ((1α,25)OH_2_ VitD3, VitD, *CD14*) or granulocyte- (DMSO, DMF, *IL8Rb*) like cells, suggesting that *SLC11A1* expression occurred late in the myelo-monocytic differentiation program. Accordingly, SLC11A1/NRAMP1 was detected in tertiary granules of neutrophils [72]. However, gene expression was not induced in response to granulocytic differentiation triggered with *all-trans* retinoic acid (ATRA), classically used for leukemia differentiation therapy [81]. The data indicate that *SLC11A1* expression is induced along either mono- or granulocytic pathways, and protein detection in the membrane of phagosomes of both types of phagocytes supports that SLC11A1 constitutes a functional marker of professional phagocytes [71,72]. The results also imply that selective pathways induce *SLC11A1* expression, presumably by activating particular (combination of) transcription factors.

### 4.2. Vitamin D and Host Defense against Tuberculosis

Vitamin D active metabolite ((1α,25)OH_2_ VitD3, VitD) binds the vitamin D receptor (VDR) and the VitD-VDR complex assembles with the retinoic X receptor (RXR, which binds 9-*cis* retinoic acid, 9-*cis* RA) [82] or with itself to form VDR-VDR homodimers [83,84]. Dimeric VDR complexes move to the nucleus where they bind to accessible VitD response elements. The VDR is ubiquitously expressed and there is growing interest to study relationships of serum levels of VitD serum precursor, 25OH VitD3, to chronic metabolic, cardiovascular, neoplastic and immunologic diseases and for considering VitD supplementation in prevention and treatment of numerous disorders [85].

Retinoids do not induce *SLC11A1* expression despite inducing granulocytic maturation (ATRA) [70] or representing the specific ligand of VDR preferred partner for heterodimerization (RXR, 9-*cis* RA) [71]. Since VDR functions also as homodimer and *NRAMP1* expression was more efficiently up-regulated specifically in the presence of VitD genomic *vs*. non-genomic agonists [71], VDR-dependent nuclear events were presumed. Gene regulation by VitD involves local chromatin remodeling events that occur in a time frame that varies with target genes [86,87,88]. *SLC11A1* expression was found slow and moderate compared to the monocyte marker *CD14*, implying perhaps an indirect process mediated by a VitD-induced factor that would bind to and regulate *SLC11A1* promoter.

The regulation of *SLC11A1* by VitD may have physiological implications since this secosteroid hormone stimulates, through VDR binding, myelopoiesis and the maturation of MNs towards macrophages with an M2—or anti-inflammatory—phenotype [89,90]. VitD also potently inhibits the maturation of dendritic cells into immunogenic antigen presenting cells [91,92,93,94,95,96]. At the same time VitD plays a key role notably through macrophages in tissue repair and peptide antimicrobial response in mammals [93,94,97,98]. Both VitD and VDR contribute to host innate resistance to infections, especially with Mtb [29].

Local productions of VitD by epithelial and immune cells as well as adipocytes exert autocrine or paracrine immunomodulating effects [85,96,99]. Hence injury or infection trigger via macrophage and keratinocyte TLR2/1 the synthesis of IL-15; IL-15 stimulates cytochrome P450, family 27, subfamily B, polypeptide 1 (CYP27B1) activity that enables transformation of 25OH VitD3 into the active form of the hormone, VitD, which in turn boosts autophagic and antimicrobial responses as well as pathogen detection [100,101]. Such autocrine production of VitD by macrophages notably allows to mount potent antimicrobial effectors that effectively counter-act the strategies of the devastating intracellular pathogens Mtb and HIV [102,103,104].

This paracrine antimicrobial role of VitD is required for IFN-γ-mediated microbicidal activity of human macrophages. T_H_1 cell-secreted IFN-γ induces VitD antimicrobial pathway (cathelicidin and β4 defensin peptides, CYP27B1 and VDR), which is similar to the response triggered by TLR2/1 ligands (e.g., Mtb-derived 19-kD triacylated lipopeptide). Both rely on monocyte secretion of IL-15 albeit through different pathways (STAT1 or MyD88). Monocytes stimulation by IFN-γ depends on the presence of sufficient VitD serum precursor as IFN-γ stimulates CYP27B1 activity and treatment with IFN-γ is inhibited by a VDR antagonist. In addition, IFN-γ-induced autophagy and phagosome maturation are VDR-dependent. A concentration superior to 45 nM of 25OH VitD3 is strictly required to observe IFN-γ—and IL-15-dependent monocyte antimicrobial responses (autophagy, antimicrobial peptides). VitD appears thus instrumental for acquired immunity to activate macrophage antimicrobial responses that overcome intracellular pathogens evasion strategy [105].

### 4.3. Regulation of SLC11A1 Proximal Promoter by Vitamin D in HL-60 Cells

In HL-60 promyelocytic cells, inducing differentiation with VitD was required to obtain upregulation of the accumulation of the endogenous *SLC11A1* mRNA in response to IFN-γ. However, once isolated from its native chromatin environment *SLC11A1* proximal promoter became directly responsive to IFN-γ treatment for two days, even though the cytokine induces little monocytic differentiation [71]. Thus *SLC11A1* may be part of the antimicrobial arsenal that is primed by VitD (or genomic agonists) and VDR, whose activity increases *SLC11A1* proximal promoter accessibility and responsiveness to transcriptional stimulation by antimicrobial factors such as IFN-γ. *SLC11A1* sequence 647 bp upstream of the ATG comprises a basal promoter element, starting 263 bp 5' of the ATG, which alone drives maximal transcriptional activity in non myeloid background (T lymphocytes and epithelial cells) and independent of VDR agonist. More distal upstream elements are required for maximal promoter expression level in promyelocytic HL-60 cells and to obtain further VDR-dependent transcriptional upregulation [71].

During differentiation various epigenetic marks decorate the genome to demarcate domains of activity that are affected in a hierarchical manner. For instance, HSC pluripotency genes get progressively turned off with the onset of differentiation, by recruiting histone deacetylases and affecting histone methylation pattern to induce formation of heterochromatin [77]. Conversely, pioneer transcription factors such as the haematopoietic lineage-determining factors (e.g., PU.1 and C/EBPs) can interact with large sets of *cis-*regulatory elements by “opening” inaccessible chromatin domains, as their binding initiate nucleosome remodeling and prime targets genes for expression in cell-type-specific manner [106]. *SLC11A1* lacks conventional TATA or CAAT box and other signals generally important for transcription activation [107]. Progressive deletion of *SLC11A1* candidate promoter region 647 bp upstream of the ATG and DNAse I footprinting analysis allowed to test the hypothesis that transcription factors gain access and bind to this region during myelo-monocytic differentiation.

*SLC11A1* major transcription start site (TSS) was mapped by 5' RACE PCR and S1 mapping in monocytic THP-1 cells and HL-60 cells differentiated into MNs, respectively [108,109]. Two *cis*-acting sites required for myelo-monocytic expression and the binding factors they recruit were identified. Double stranded DNA probes corresponding to footprints protected *in vitro* from DNAse I digestion after incubation with nuclear extracts from differentiated HL-60 cells were characterized by electromobility shift and supershift assays. The candidate *cis* elements were tested *in vitro* by site-directed mutagenesis and the effect of linker-mutations was verified by reporter assays after transfection or co-transfection of promoter constructs with selected transcription factors. Interaction of the candidate factor with the promoter area containing the site defined was verified by chromatin immuno-precipitation (ChIP) assay. The results showed that *SLC11A1* major TSS is adjacent to a 5' C/EBP binding site, which is required for transcriptional activation during monocytic and granulocytic differentiation, and within an area bound by C/EBPα in promyelocytic cells and by C/EBPβ beginning 24 h after induction of differentiation with VitD. Site-directed mutagenesis of this C/EBP binding site also abrogated transcriptional activity of the promoter in non myeloid background implying it is required for recruiting the basal transcription complex. A binding site for the Specificity protein 1 (Sp1), which transactivates *SLC11A1* promoter *in vivo*, was delineated in the more upstream region of the proximal promoter that is required for myeloid expression [109].

Several members of the C/EBP family of bZIP transcription factors have important roles in myeloid development, and in macrophage in particular, as C/EBPα , C/EBPβ and C/EBPδ represent three of the 18 regulators most frequently represented in the 14 modules enriched for macrophage-related gene signatures [25]. The induction of C/EBPβ expression by another bZIP family transcription factor, the cAMP responsive element-binding protein (CREB), results in specific upregulation of M2-associated genes in response to LPS; C/EBPβ is also required for muscle repair after injury, indicating that the CREB-C/EBPβ axis is crucial for M2 macrophage polarization [110]. C/EBPβ contributes in general to regulate myeloid proliferation and differentiation, the expression of inflammatory genes as well as host defense against microbial infections [111].

C/EBPβ (a.k.a. NF-IL6) is a key regulator of monocytic cells development [111]. It serves as “pioneer factor” providing the sequence specificity to endow general chromatin remodelling complexes, including the “switch/sucrose nonfermentable” (SWI/SNF) nucleosome remodelling complex, with transcription enhancer-specific roles [112]. More generally C/EBPs form a functional interface with the transcription factor PU.1 member of the E26 transformation specific (ETS) family, which is expressed at high levels to modulate the regulation of cell-specific macrophage functions [110]. Hence, PU.1 and C/EBP α/β convert fibroblasts into macrophage-like cells [113] and determine the ratio of myelo-monocytic to erythrocytic cells committing from common myeloid progenitors (CMP, Figure 2A) [76]. C/EBPα is indispensable for early granulocyte development, as the balance between PU.1 and C/EBPα controls the bifurcation between the mono- and granulocytic pathways, and C/EBPα upregulates the transcription of several granulocyte-specific factors [78]. All the other C/EBP isoforms also contribute to the transcriptional regulation of granulocytic genes later in development after the promyelocyte stage [78]. Given their prominent and complementary roles in myelo-monocytic differentiations it seems fitting that *SLC11A1* expression in professional phagocytes is controlled by C/EBPs.

Regarding macrophages, *SLC11A1/NRAMP1* gene expression was detected not only in pro-inflammatory (M1) but also anti-inflammatory (M2) macrophages [114,115]. Moreover, Nramp1 activity was also found to contribute to erythrocyte iron recycling after inducing hemolytic anemia [73] and the protein localizes to the membrane of phagosomes containing apoptotic erythrocytes [74]. C/EBPβ expression is stimulated by and mediates the monocytic differentiation program induced by VitD, known to skew macrophage phenotype towards M2 activity [89,90]. The VDR, which mediates VitD genomic actions [116] is another key determinant of human myelo-monocytopoiesis [76]. At homeostasis, macrophage M2 polarization is maintained notably through recycling of apoptotic cells by phagocytosis. Apoptotic cells liberate various mediators including oxysterols and fatty acids. These lipid mediators are agonists of the LXRs and PPARs, nuclear receptors related to VDR that coordinate efficient engulfment and anti-inflammatory metabolic recycling of apoptotic cells [117,118,119]. These data suggest that C/EBPβ control of *NRAMP1* expression may relate to steady state activities of tissue macrophages and maintenance of tissue integrity.

The contribution of the Specificity protein 1 (Sp1) to regulate *SLC11A1* transcription may support this view because Sp1 has been widely associated with basal levels of constitutive expression, including at various myeloid promoters. Despite being widely expressed the transcription factor Sp1 can regulate the expression of tissue-specific genes through combinatorial effects with a limited number of key transcription factors [120]. Hence, Sp1 is essential for myeloid-specific promoter activity of *C/EBPδ* , the complement and pattern recognition receptors *CD11b* and *CD14*, respectively, *lactoferrin* and the *Myeloid Elf-1 like Factor* (*MEF*) which codes for an ETS protein that activates the promoters of myeloid effectors and stimulators such as the granulocyte macrophage colony-stimulating factor, interleukin-3, lysozyme, human beta defensin-2 and perforin [121,122,123,124,125,126]. As previously observed in other myeloid promoters [120,124,126] several Sp1 binding sites were found in *SLC11A1* proximal promoter upstream region which is required to confer myeloid specific expression and VDR-dependent upregulation. Mutating one binding site for Sp1 had less impact than abrogating *SLC11A1* TSS C/EBPα/β site [71,109]. Hence, it is speculated that Sp1 contributes to *SLC11A1* myeloid-specific expression through combinatorial interactions including transcription factors key for myelomocytic differentiation such as C/EBPβ.

### 4.4. Transcriptional and Post-Transcriptional Determinants of SLC11A1 Expression in Macrophage-Like HL-60 Cells

Another *cis* element key for *SLC11A1* expression along macrophage-like differentiation induced with PMA was recently characterized. PMA induces *SLC11A1* transcriptional activation in HL-60 cells and the mRNA is stabilized by the interaction of a 3' AU-rich element (ARE) with a ubiquitously expressed RNA-binding protein, Hu antigen R (HuR). PMA-induced migration of HuR from the nucleus to the cytoplasm paralleled increased binding of HuR to *SLC11A1* ARE and the accumulation of transcript. Overexpression of HuR in HL-60 cells stabilized *SLC11A1* mRNA levels induced after 3 days PMA treatment, whereas siRNA HuR knockdown limited both *SLC11A1* transcript and protein accumulation [127]. The RNA recognition motifs of HuR interact with mRNAs involved in cell cycle, cell death and differentiation, immunity, and inflammation. HuR can either stabilize or promote destabilization of its targets, through interactions involving suppressive RNA-binding proteins (RBPs), microRNAs, and associated factors. Hence *HuR-null* macrophages show exacerbations in the biosynthesis of inflammatory cytokines and chemokines; HuR is essential for balancing proinflammatory response and homeostatic regulation to control the extent of the inflammatory response [128]. Given the pro-inflammatory potential of SLC11A1/NRAMP1 activity it should be interesting to further analyze how macrophage activation phenotype may modulate HuR-dependent SLC11A1 protein expression level.

Progress in understanding molecular mechanisms of *SLC11A1* transcriptional regulation during macrophage-like differentiation of HL-60 cells pointed at a PMA-responsive element, which was located by nested deletions of a reporter construct including the upstream myeloid-specific region. The PMA responsive element is adjacent in 5' to the (GT)_n_ repeat sequence that is converted into Z-DNA conformation and which contains two binding sites for the HIF-1α/ARNT heterodimer [49,129] (Section 3.3). Macrophage-like differentiation induces recruitment to the PMA-responsive element of the Activator protein-1 (AP-1)-like factor ATF-3/JunB, together with β-actin and BRG1, which were revealed by DNA affinity pulldown assays using double stranded oligonucleotide probes and antibodies against ATF-3, JunB, β-actin and the actin dependent regulator of chromatin BRG1, as well as ChIP assays. ATF-3 siRNA knockdown prior to DNA affinity pulldown assays targeting BRG1 and β-actin indicated that the presence of ATF-3 was necessary to recruit BRG1 and β-actin to the DNA probe, and immunoprecipitation assays confirmed that the three factors form a complex [129].

In the days following PMA-induced differentiation of HL-60 cells, β-actin translocates from the cytoplasm to the nucleus in a process that requires p38 MAPK activity [129]. ChIP-on-chip assays showed the genome-wide map of nuclear β-actin binding to promoters of genes involved in diverse functions (cell growth and differentiation, ion transport, adaptive and immune responses and signaling). β-actin binding was correlated with recruitment of RNA polymerase II (RNA Pol II) at six promoters including *SLC11A1* [130]. siRNA knockdown of β-actin, ATF-3 or BRG1 reduced *SLC11A1* transcription while co-transfection assay showed additive effects of these factors to activate *SLC11A1* transcription. The formation of Z-DNA was detected by transfection of a *SLC11A1* promoter construct in differentiated HL-60 cells, which were then cross-linked with formaldehyde and permeabilized before adding a fusion protein that binds Z-DNA structure and catalyzes double-stranded DNA cleavages in the vicinity. Detection of the cleavage sites by LM-PCR showed digestion products in cells differentiated with PMA that was reduced by siRNA BRG1 knockdown, demonstrating that this ATPase subunit of the SWI/SNF chromatin remodeling complex plays a critical role in Z-DNA formation and SWI/SNF-mediated transcriptional regulation [116].

The Activating transcription factor 3 (ATF3) is a member of the ATF/cyclic AMP responsive element binding family (CREB) family of transcription factors. ATF3 is expressed in a number of splice variants and interacts with multiple partners to form dimeric complexes, acting either as repressor or activator of various genes. For instance in Toll-like receptor (TLR)-activated macrophages, ATF3 recruits the histone deacetylase 1 (HDAC1) to the promoters of genes encoding cytokines or involved in metabolic and inflammatory responses, exerting negative regulation by epigenetic modification [131]. ATF3 is generally recognized as a repressor that is transcriptionally up-regulated by TLR signaling (TLR2/6, TLR3, TLR4, TLR9) and which regulates negatively the transcription of pro-inflammatory cytokines, such as interleukin (IL)-6 and IL-12p40, notably by recruiting HDAC1 and antagonizing transcription of target genes. Accordingly, *atf3* deficiency induces overt susceptibility to endotoxic shock induced death [132]. However, ATF3 has the ability to interact with a number of transcription factors and other bZIP-containing proteins including AP-1 (c-Jun, JunB, JunD), C/EBP or MAF families of proteins, and depending on the promoter context, these heterodimers can act either as repressors or activators so that the role of ATF3 as transcriptional repressor or activator cannot be generalized [132]. In addition, microsatellite allelic variation can affect the binding of ATF-3 and c-Jun, as shown for *SLC11A1* proximal promoter [50].

The recruitment of β-actin to *SLC11A1* promoter stimulates transcriptional activation [130]. The basic property of β-actin is to polymerise into helical filament which can be used to produce force inside the cell. Actin in a nucleus (nuclear actin) is a component of many important chromatin remodeling complexes and it has been connected to most steps of the transcription process by all three RNA polymerases from the regulation of transcription initiation to the processing of pre-mRNAs [133]. It has been proposed that β-actin and actin-related proteins produce the maximum ATPase activity of SWI/SNF and favor stable association between chromatin and the remodeling complex [134]. Cell differentiation is normally a non-reversible process that silences transcriptional programs that would otherwise reactivate genes which were active in stem cells. But abundant actin in the *Xenopus* oocyte nucleus enables to reactivate heterologous embryonic genes, indicating that nuclear actin allows transcriptional activation by de-repressing silenced genes and this regulation is an evolutionary conserved feature [135]. Accordingly, ATF-3/JunB binding would allow the recruitment of β-actin and BRG1 to *SLC11A1* promoter, enhancing the ATPase activity of the SWI/SNF chromatin remodeling complex to create a Z-DNA structure that may facilitate interaction with HIF-1α/ARNT heterodimers and the activation of *SLC11A1* transcription in the days following induction of differentiation with PMA.

*SLC11A1* transcriptional activation during VitD and PMA differentiation followed similar time frames. Since β-actin translocates from the cytoplasm to the nucleus after two days differentiation induced with PMA and, because C/EBPs can also recruit the SWI/SNF chromatin remodeling complex, it would be interesting to determine if VitD-induced *SLC11A1* transcription depends also on β-actin stimulation. Alternatively the mode of transcriptional activation could vary depending on the developmental program since C/EBPα was shown to bind *SLC11A1* TSS in undifferentiated promyelocytic cells. In contrast, *NRAMP1* Z-DNA interaction with ATF-3 and HIF-1α/ARNT were evidenced in cells more advanced along the monocytic differentiation pathway, and in response to typical M1 macrophage activating stimuli such as LPS and IFN-γ [136]. Yet both HIF-1α and C/EBPs contribute to regulate cell energy metabolism, which is affected by macrophage polarization, and recent evidence suggested that HIF-1α and C/EBPα can interact and induce reciprocal functional changes [137]. In monocytic U937 cells, C/EBPα directly up-regulates the transcriptional expression of *galectin-1*, also regulated by HIF-1α, which interacts with and enhances the transcriptional activity of C/EBPα [138].

Induction of *SLC11A1* transcription early in the myelo-monocytic development program may require as well recruitment of the co-activator Mediator multiprotein complex, which acts as molecular bridge between promoter-bound activators and the core transcriptional machinery. Both C⁄EBPβ and Sp1 can bind elements of this complex which may serve to integrate signaling toward the transcriptional machinery during cell differentiation. Another possibility would consist in recruiting coactivator complexes with HAT activities such as p300; histone acetylation may either provide binding surfaces for other activator proteins or facilitate chromatin decondensation to increase accessibility to the transcription machinery. However it might be equally important to consider as well the possible contribution of distal elements which may provide critical signals to recruit the basal transcription machinery [139,140,141,142].

## 5. Delineation of *SLC11A1* Distal Elements Mobilized During Myelo-Monocytic Development

HSC differentiation results from a stepwise process that produces cells demonstrating various degrees of lineage potential until reaching a stage of committed progenitor, which yields only one lineage through a multistage process producing mature cells. Because most eukaryotic DNA is packaged in closed, tightly packed chromatin conformation (heterochromatin), chromatin structure has an important role at the local and chromosomal levels in controlling hematopoieteic gene expression by regulating accessibility to the transcription machinery [77]. The corner stone of gene activity is the binding of transcription factors and co-regulators to specific DNA sites. Models of myeloid-specific gene regulation indicate that local, differentiation-induced binding of *trans*-factors leads to a dynamic primed state and partial chromatin remodeling, which then evolves from resting to induced state [143,144]. Regulation of myeloid differentiation by HDAC or HAT inhibitors [145,146] suggests that genes primed during differentiation are sensitive to a dynamic state of acetylation [142,147,148].

Using the University of California Santa Cruz (UCSC) Genome Browser [149] to visualize the available genomic data that were produced by high throughput unbiased approaches through the Encyclopedia of DNA Elements (ENCODE) [150,151,152], whose goal is to identify all functional elements in the human genome, allows longitudinal analysis of the distribution of DNA and histone marks at a selected locus, considering discrete stages along the relevant hematopoietic differentiation pathway. These data may thus be useful to delineate regulatory elements that are mobilized for developmental control of gene expression, and suggest candidate distal regions that may contribute to regulate *SLC11A1* expression or support results previously obtained following hypothesis-driven approaches.

### 5.1. Physical Organization of SLC11A1 Locus

**Figure 3:**
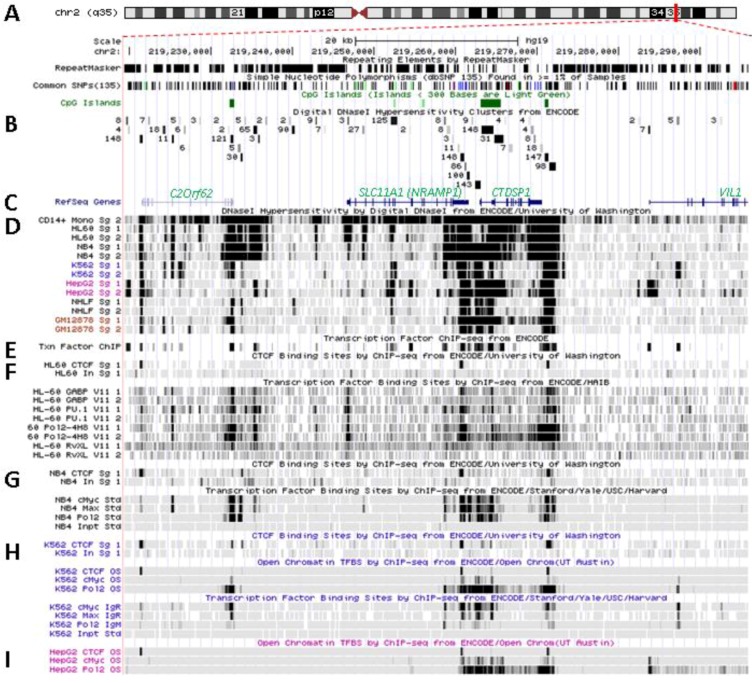
Transcriptional activity at *SLC11A1* locus 2q35. (**A**)University of California Santa Cruz (UCSC) genome browser [149] visualization of chromosome 2 and blow-up at q35 showing coordinates, scale (20 kb) and few basic sequence elements: common repeats and single nucleotide polymorphisms (freq >1%), CpG Islands (color code >300 bp, darker). (**B**) ENCODE DNAse I hypersensitivity clusters reported for 148 cell-types [152], indicating regions of nucleosome remodeling and suggesting enhancer or promoter activity. (**C**) RefSeq genes. (**D**) ENCODE DNAse I hypersensitivity raw signals from CD14^+^ MNs, APLs (HL-60, NB4), CML (K-562), Hepatoma (HepG2), Fibroblast (NHLF) and B lymphoblast (12878). (**E**) Compiled ChIP-Seq transcription factor data from ENCODE [150] (detailed in Figure 5D). (**F**–**I**) Raw ChIP-Seq data targeting the transcription factors indicated in the cell-types APL, HL-60 (**F**) and NB4 (**G**), CML, K-562 (**H**), and the non-myeloid Hepatoma HepG2 (**I**).

*SLC11A1/NRAMP1* localizes at 2q35 in a densely populated chromosomal region between the gene encoding the unknown Orf *C2ORF62*, upstream, and *CTDSP1*, coding for the CTD (carboxy-terminal domain, RNA Pol II, polypeptide A) small phosphatase 1, adjacent to *SLC11A1* 3' end (Figure 3A). The three genes have the same orientation coded by plus strand and are apparently free of miRNA regulatory sites (TargetScan, not shown). Few CpG islands are present in regions free of repeated sequence. These are 0.5–2 kilobase (kb) DNA fragments rich in CpG dinucleotides, usually constitutively protected from methylation by specific transcription factors such as Sp1, and which adopt an accessible conformation for potential regulatory factors [136]. One is found at the 3' end of *C2ORF62* and two delimitate *CTDSP1*; three smaller elements (<300 bp) are present within *SLC11A1*, between exons 7–8 and 10–11, while a few CpG dinucleotides were found mainly clustered in the basal proximal promoter region around the TSS [109].

A single predicted transcription start site (SwitchGear Genomics) locates 41 bp downstream the major TSS mapped experimentally [108,109]. *SLC11A1* TSS established by independent approaches maps 148 bp 5' of the translational initiation codon. The proximal promoter region of *SLC11A1* and those of flanking genes display sequence conservation among vertebrates, together with exons and 3' UTR (*SLC11A1* and *CTDSP1*); some areas in *SLC11A1* putative distal promoter also show sequence conservation suggesting that some *cis* regulatory elements might have been preserved across species.

### 5.2. In Situ DNAse I Footprinting and Transcription Factor-Specific ChIP-Seq Studies

*SLC11A1* locus displays several areas that correspond to hypersensitive sites to DNAse I digestion in intact nuclei among 148 cell types, which were identified by direct sequencing of the ends of DNAse I “double-hit” fragments (UW DNAse I HS, Figure 3B) [153]. The resulting sequencing footprints correspond to individual DNAse I cutting events that indicate open chromatin regions and correlate with active transcription. Some of the DNAse I footprints were detected in every cell type tested whereas others were found only in myelo-monocytic cells: CD14^+^ MNs, HL-60 and NB4 promyelocytes; some are also present in the megakaryocytic lineage (K-562) and not in hepatocyte, lymphocyte or fibroblast cells for instance (HepG2, GM12878 and NHLF, respectively, Figure 3D). Several sequence segments sensitive to DNAse I digestion (Figure 3B,D) do overlap with small chromosome fragments delineated independently by chromatin immunoprecipitation assay coupled to massively parallel sequence-based detection (ChIP-Seq) which targeted specific transcription factors interacting with DNA either directly or indirectly, in selected cellular background (SYDH TFBS, Figure 3E).

Substantial overlap between the sequence segments obtained by either enzymatic digestion of open chromatin or mechanical fragmentation based on specific interaction with nuclear proteins hence suggests candidate regulatory factors *trans-*interacting with potential *cis* acting elements [154]. For instance, three DNAse I footprints around *SLC11A1* gene and found in all 148 cell types tested (Figure 3B) overlap with DNA fragments recovered by ChIP-Seq that bound ubiquitous factors, e.g., the locus insulator CCCTC-binding factor (CTCF) and RNA Pol II (Figure 3E). Insulator elements exert a dual function by preventing the spread of heterochromatin (barrier function) and transcriptional enhancers from activating unrelated promoters (enhancer blocking) [139].

Among the 50 DNAse I footprints examined over the selected 76,543 bp DNA fragment that spans from *C2ORF62* to *VIL* (Figure 3B), 14 signals were absent from myelo-monocytic nuclear extracts, including nine found in *C2ORF62* or *VIL*; seven footprints were found only in myelo-monocytic cells, and for seven others half of positive extracts were from the myelo-monocytic lineage.

#### 5.2.1. Myelo-Monocytic-Specific Signals

**Figure 4:**
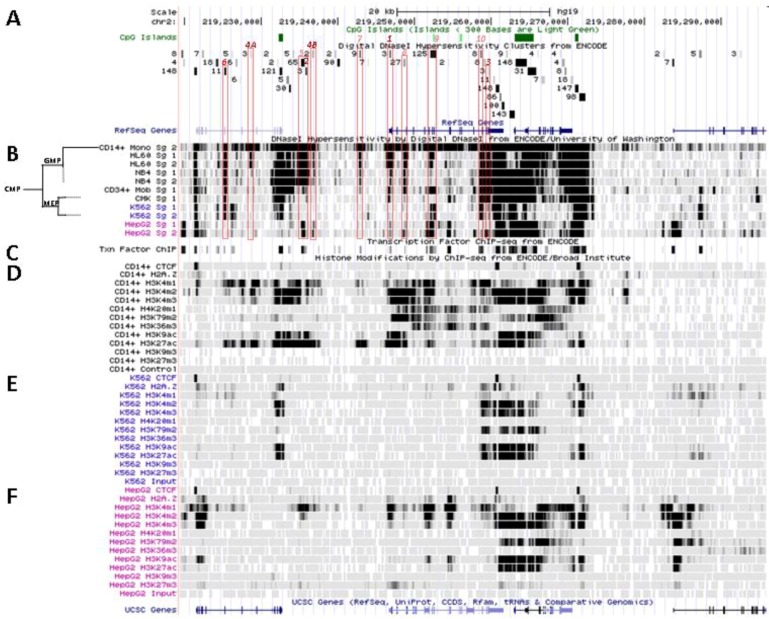
Nucleosomal remodeling at *SLC11A1* locus in mature CD14^+^ monocytes. (**A**) 2q35 coordinates, CpG islands, DNAse I hypersensitivity clusters and RefSeq genes as in Figure 3; red boxes indicate the 11 DNAse I hypersensitivity clusters highly represented in myelo-monocytic cell-types. (**B**) Raw signals from DNAse I hypersensitivity in cell-type representing stages along the myeloid pathway hierarchy with segregation of the sister megakaryo-erythrocytic and myelo-monocytic lineages (cf Figure 2A). (**C**) ENCODE transcription factor ChIP-Seq data as in Figure 3. (**D**–**F**) ChIP-Seq raw data for CTCF, H2A.Z and histone modification marks associated with transcriptional activity (H3K4me1-3, H4K20me1 , H3K79me2, H3K36me3, H3K9ac, H3K27ac) or inhibition of transcription (H3K9me3, H3K27me3) in myeloid (CD14^+^ MNs, **D**, K-562, **E**) and non-myeloid (HepG2, **F**) background.

The interval covering *SLC11A1* 5' promoter and coding regions contains most of DNAse I footprints that were evidenced specifically with myelo-monocytic nuclear extracts. These areas are found between stretches of repeated sequences and comprise potential *cis* acting regulatory elements likely to contribute to myelomonocytic-specific expression. Hence, C/EBP transcription factors bind to *SLC11A1* TSS within the chromatin of promyelocytic HL-60 cells and C/EBPβ binding is necessary for differentiation-induced transcriptional activation [109]. Accordingly, this C/EBP site is part of a strong DNAse I footprint that was obtained in human HL-60 cells and CD14^+^ MNs but not in 146 other cellular background tested (Figure 3B,D). The 170 bp DNAse I sensitive fragment (Footprint #1 in Figure 4A,B) is delimited more or less closely by two *cis* elements previously defined, the 5' Sp1 binding site E10 and the C/EBP α/β site adjacent to *SLC11A1* TSS, 5 bp upstream of the footprint 3' end (Section 4.3) [109].

**Figure 5:**
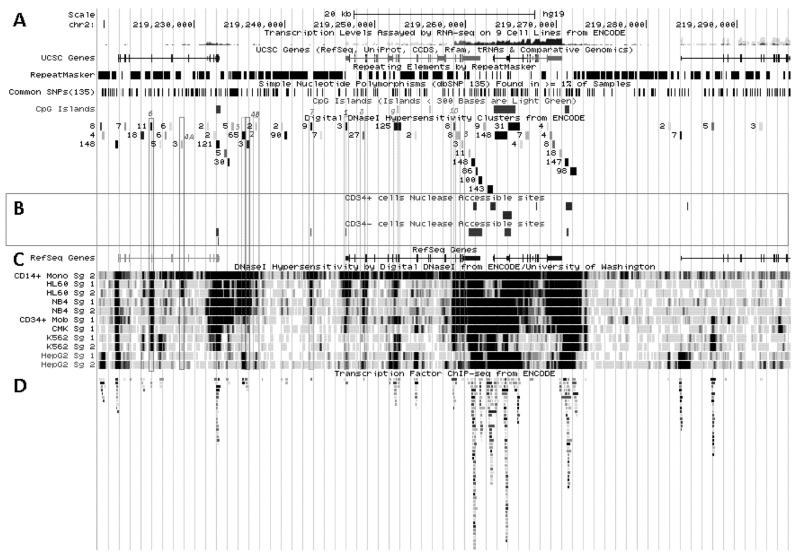
*SLC11A1* transcriptional activation during monocytic differentiation. (**A**) 2q35 coordinates and scale, and summary of RNA-Seq transcript levels from ENCODE cell lines (K-562, purple, HepG2, green, NHLF, pink and GM12878, red). UCSC genes; repeated elements; common SNPs; CpG islands and ENCODE DNAse I hypersensitivity clusters, as in Figure 3A ,B. (**B**) Endonuclease accessible areas indicating locally open chromatin in CD34^+^ HSPC and their monocytic progeny CD34^−^ CD13^+^ CD33^+^ (EIO-JCVI) [158]. (**C**) RefSeq genes and raw signals of DNAse I hypersensitivity in cell lines representing various stages of myelopoiesis. (**D**) ENCODE inventory of all the transcription factors reported so far associated with *SLC11A1* locus by ChIP-Seq.

This area is also covered by a larger DNA segment that was reported by ChIP-Seq analysis of HepG2 cells, after immuno-precipitation using antibodies specific for C/EBPβ followed by high throughput sequencing, suggesting that in HepG2 nuclei *SLC11A1* TSS may also bind C/EBPβ. However, in HepG2 cells the 5' part of the gene also carries marks of gene silencing that were revealed in independent analyses (H3K27me3; UW Histone, Broad Histone, end of Section 6). Accordingly, *SLC11A1* expression might be prevented [155] despite some binding of C/EBPβ at *SLC11A1* TSS. This interpretation is supported by detection of low level *SLC11A1* transcription in HepG2 cells (ENCODE Caltech RNA-Seq; Figure 5A). These data suggest that the C/EBPα/β binding site required for *SLC11A1* transcription is accessible and activated only in the chromatin context of terminal myelo-monocytic differentiation.

One of the signals found selectively in myelo-monocytic nuclei constitutes the C/EBPα/β binding site at *SLC11A1* TSS (Footprint #1, Figure 4A ,B); another located ~11 kb upstream represents a strong candidate C/EBPβ binding site (Footprint #2, Figure 4A,B), and a footprint at the 3' end of the gene 13 kb past the TSS may correspond to a binding site identified in K-562 cells for the E74-like factor 1 (ELF-1), another ETS-related transcription factor (Footprint #3, Figure 4A,B).

These footprints at sites distant from *SLC11A1* TSS were reported in promyelocytic cells only (NB4, HL-60; UW DNAse I HS). ELF-1 controls the expression of multiple essential haematopoietic regulators; its downregulation is necessary for erythrocyte differentiation [156] and it was involved in the regulation of Fc receptor gamma-chain gene expression in macrophages [157]. Importantly, *SLC11A1* candidate functional polymorphism D543N (Section 3.2) is carried by DNA fragments that were either digested by DNAse I or pulled-down by ChIP-Seq targeting ELF-1 transcription factor, implying that exon XV may contribute to regulatory functions. This result also warrants re-interpretation of the possible function of this non-synonymous polymorphism, which may either cause a missense mutation or affect DNA-protein interactions.

**Figure 6:**
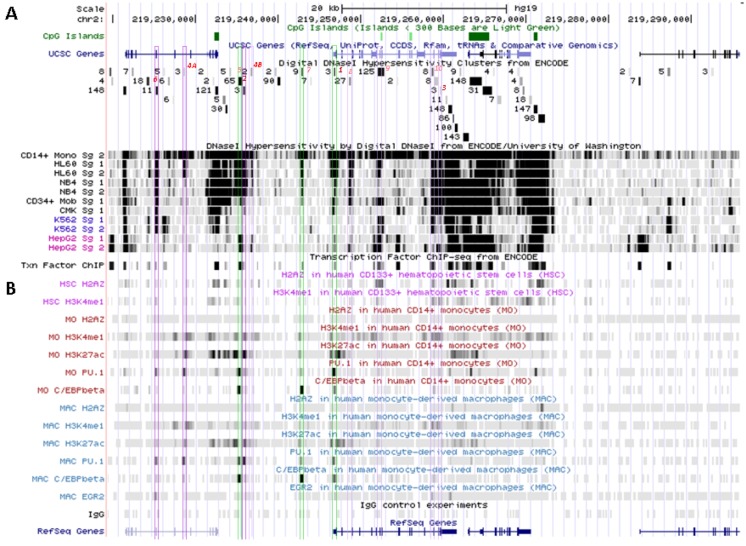
Mobilization of *SLC11A1* 5' large enhancer in mononuclear professional phagocytes. (**A**) 2q35 coordinates and scale, CpG islands, UCSC genes, ENCODE DNAse I hypersensitivity clusters and transcription factor ChIP-Seq, as in Figure 5. (**B**) UCSC browser visualization of raw Chip-Seq data showing *SLC11A1* locus epigenetic enhancer signature (H2A.Z, H3K4me1 and H3K27ac) and DNA association with the transcription factors PU.1, C/EBPβ and EGR-2 key for terminal myelomonocytic differentiation [80]. HSC, hematopoietic stem cells, MO, monocytes, MAC, monocyte-derived macrophages. Color boxes indicate the 11 DNAse I hypersensitive sites identified in myelo-monocytic cell-types (Section 5.2); in green, the predicted C/EBPβ binding sites that actually bind this factor in mature myelo-monocytic cells.

No candidate transcription factor has yet been identified by ENCODE ChIP-Seq analyses for the remaining four DNAse I footprints selectively found in myelo-monocytic background (CD14^+^ MNs, HL-60 cells, ~18 and 10 kb upstream of *SLC11A1* TSS, Footprints #4_A_ and #4_B_, Figure 4A,B, or NB4 cells, ~15.5, 14.5 kb upstream of the TSS). However, independent search for monocytic enhancer signatures indicates that Footprint #4_A_ corresponds to an area that associates with PU.1 in CD14^+^ MNs and macrophage derived from them (MDMs; Figure 6) [80].

*SLC11A1* upstream potential C/EBPβ binding site is delineated by the overlap between a DNAse I footprint (Footprint #2, Figure 4A,B) and a DNA fragment pulled down in ChIP-Seq directed either at C/EBPβ or the histone deacetylase-2 (HDAC2, Figure 4C, Figure 5D). This candidate C/EBPβ binding site is flanked by a downstream predicted site for a transcription factor of the mammary cell-activating factor (MAF) family; these two sites are part of a 290 bp DNAse I sensitive fragment while the specific ChIP-Seq DNA segments display 100 bp increment. This area also matches a strong PU.1 binding site only in MDMs (Figure 6B) supporting a possible regulatory role.

Another hypothetical C/EBPα/β site further upstream is flanked by a 3' binding site for the Signal transducer and activator of transcription 3 (STAT3), which are predicted on similar grounds (DNA sequence fragments hypersentive to DNAse I matching those pulled by ChIP-Seq assay): both candidate sites are covered by a 410 bp genomic footprint; the DNA fragments pulled down by ChIP-Seq share the 5' boundary but STAT3-specific segments extend 150 bp further in 3'. This upstream C/EBP site is equally associated with C/EBPβ and PU.1 both in CD14^+^ MNs and MDMs (Figure 6) supporting a role in terminal myelo-monocytic differentiation [80].

These two predicted C/EBP sites ~400 bp distant differ in occurrence among cell types. The more upstream region that can interact with STAT3 and C/EBPβ was sensitive to DNAse I in 65 cell types, giving intense signals in MNs, HL-60 and NB4 (by decreasing order; Footprint #5, Figure 4A,B). In contrast the downstream DNAse I sensitive region, bound by MAFK, HDAC2 and C/EBPβ in HepG2 cells, was detected only in promyelocytic cells, with by decreasing order NB4 and HL-60 cells. These differences relate as well to variations in nucleosomal histone mark composition, which indicate the more 5' element as rather common whereas downstream sites present more cell-specific patterns (Section 6.2). Promyelocytic cells correspond to an early bi-potential stage of differentiation toward either monocytic or granulocytic lineages (Figure 2A) [76]. As the C/EBPα/β site at the TSS showed weak DNAse I sensitivity in NB4 background (Footprint #1, Figure 4A,B) it is likely that *SLC11A1* expression is reduced in these cells, consistent with observation of limited histone modifications (UW Histone, Section 6.3.3).

STAT3 and C/EBPβ have complementary roles supporting host defense against infection as both are IL-6-regulated transcription factors [159]. C/EBPβ also stimulates IL-6 expression induced by IL-1 [160] whereas STAT3 mediates IL-6 signal transduction and the acute phase response (APR) [161]; STAT3 also stimulates hepatic hepcidin expression [162]. The proximity of STAT3 and C/EBPβ upstream binding sites could create a regulatory hub to enhance *NRAMP1* transcription in response to inflammatory stimuli. In addition, the MAFK site predicted adjacent to the downstream myeloid-specific C/EBPβ binding site may contribute to regulate expression in non-inflammatory conditions: MAF transcription factors are AP-1 family members which, similarly to C/EBPβ [111], can function as activator (long isoforms) or repressors (MAFK and other small MAFs), depending on the presence of a *N*-terminal transactivator domain that allows to recruit co-activators such as p300 and CRE binding protein (CREBP) [163].

The long isoform MAFB induces monocytic differentiation [164] and prevents self-renewal of differentiated functional M2 tissue macrophages [165], which might be antagonized by IL-4 signaling [166]. *MAFK* is frequently a target of chromosomal translocations associated with acute myeloid leukemia (AML, Inv(16)). It encodes a short polypeptide that, besides acting as a dominant negative protein antagonizing large MAF proteins, can also regulate the transcription of heme oxygenase (HO-1) [167]. Induced in response to cellular stress MAFK can heterodimerize with members of the Cap “n” Collar (CNC) transcription factor family that includes nuclear factor-erythroid 2-related factor 2 (NRF2) and the more distantly related BACH (BTB (broad-complex, tramtrack and bric-a-brac) and CNC-type) 1 and 2. The functional balance between BACH1, a heme-dependent transcriptional repressor [168], and the redox sensitive transcriptional activator NRF2 [169] allows M2 macrophages to exert anti-inflammatory regulatory roles [170,171]. Also, Maf, MafB, Nrf2 and Nfe2 and Bach1 represent five of the 18 regulators most frequently represented in the 14 modules enriched for macrophage-related gene signatures [25] implying that they may contribute to regulate *SLC11A1* myelo-monocytic expression.

#### 5.2.2. Signals Enriched in Myelo-Monocytic Cells

Candidate transcription factors are suggested for one of them: a 210 bp DNAse I sensitive fragment closely overlaps with three DNA segments recovered independently by ChIP-Seq which bound PU.1, EGR-1 and USF1 in the chromatin of megakaryocytic K-562 cells. The megakaryocytic lineage constitutes a myeloid sister group of the myelo-monocytic lineage (Figure 2A) [76]. This DNAse I footprint (#6, Figure 4A,B) was detected in promyelocytic cells modeling the bi-potential stage of differentiation toward either monocytic or granulocytic lineages [76] and in MNs (Figure 2). PU.1 binding was also detected both in CD14^+^ MNs and MDMs, albeit with moderate intensity, and EGR-2 co-binding was reported in MDMs as well (Figure 6) [80].

The upstream stimulatory factor (USF1) is a ubiquitous c-Myc-related regulatory factor required for M6PR promoter activity [172]. The early growth response-1 (EGR-1) regulatory factor belongs to the C2H2-type zinc-finger protein family; it is responsible for the expression of Tissue factor in LPS-stimulated macrophages and is inhibited by the anti-inflammatory cytokine IL-10 [173]. The macrophage fate-determining PU.1 is a key developmental transcription factor that sets a chromatin context enabling the activity of ubiquitous transcription factors activated by inflammatory stimuli, such as NF-kB, AP-1, and interferon regulatory factors (IRFs) [76,174].

This candidate 5' distal myeloid *cis-*acting determinant (~21.5 kb upstream *SLC11A1* TSS, Footprint #6, Figure 4A ,B) locates downstream a strong binding signal of the locus insulator CTCF detected in all nuclear extracts (see below Broad Histone, Section 6.2 and Figure 3F–I). Such sequence-specific zinc finger transcription factor can block enhancer activity and the spreading of chromatin structure [175]. The DNAse I footprint and transcription factor ChIP-Seq data suggest that this potential 5' distal myeloid *cis* element may contribute to regulate *SLC11A1* expression during myelo-monocytic development. Presence at the 3' end of *SLC11A1* Orf of another DNAse I footprint found in myelo-monocytic extracts only (Footprint #3, Figure 4A ,B) supports this proposition. The 3' footprint overlaps with a DNA fragment pulled-down by anti-ELF-1 ChIP-Seq analysis in K-562 cells. The corresponding predicted ELF-1 site locates ~1 kb upstream a CTCF insulator site revealed by ChIP-Seq in many cell types, including K-562 (e.g., Figure 3F–I), and near a DNA polymorphism that affects innate resistance/susceptibility to TB (Section 3.2).

ELF-1 and PU.1 belong to the same family and ELF-1 is also known to contribute enhancer activities [158,176]. The corresponding footprints were detected only in cells representing promyelocytic bi-potential progenitors (HL-60, NB4); though both distal from *SLC11A1* TSS they sit within a ~34.6 kb interval defined by two major CTCF insulator sites (Figure 3F–I). In addition, K-562 chromatin appears relatively open between these CTCF sites (see below). It is thus possible to suggest that *SLC11A1* distal *cis* elements predicted to bind ETS family members such as PU.1 (upstream) and ELF-1 (downstream) may contribute to regulate gene transcription during myeloid differentiation leading to mature myelo-monocytic cells. This proposition is supported by observing that both these elements are part of areas decorated by histone modification marks which correlate with cell-type specific regulatory elements (Section 6.2) [154].

Based on currently known DNA-protein interactions at *SLC11A1* locus it is suggested that DNAse I footprints detected predominantly in CD14^+^ MNs reflect sites that are active in mature cells, including the TSS and two regions selectively footprinted in myelo-monocytic cells (MNs and HL-60) ~18 and 10 kb upstream *SLC11A1* TSS. Indeed, Footprint #4_A_ likely represents a PU.1 binding site in terminally differentiated monocytic cells (Figure 6). Regions more strongly footprinted in nuclei of promyelocytic progenitors (NB4, HL-60) may point at sites which are mobilized along myelo-monocytic development, including four other DNAse I footprints observed selectively in myelo-monocytic cells. Footprints mainly found in myelo-monocytic extracts (~50% signals) were in majority obtained using NB4 and HL-60 nuclei though four of them were detected as well in CD14^+^ MNs. However, three lie downstream *SLC11A1* 3' CTCF site and their significance is unclear.

#### 5.2.3. Other Strong Signals in Myelo-Monocytic Cells

Few other DNAse I footprints within the CTCF boundaries of *SLC11A1* locus may be considered important for controlling developmental expression based on signal intensity obtained with myelo-monocytic nuclei. Among these, a strong signal ~4 kb upstream *SLC11A1* TSS that shows myelo-monocytic selectivity (3/9) was detected in CD14^+^ MNs, and to lesser extent in HL-60 cells (Footprint #7, Figure 4A ,B). The DNA fragment identified matches an area that interacts with C/EBPβ in HepG2 cells (Figure 4C and Figure 5D), similarly to the C/EBPα/β sites at *SLC11A1* TSS and the double site located ~11 kb upstream, hence suggesting a third possible location for interaction between *SLC11A1* promoter and C/EBP factors. Accordingly, strong binding of C/EBPβ was detected at this site both in CD14^+^ MNs and MDMs, together with weaker signal for PU.1 binding (Figure 6), indicating potential enhancer activity [80].

About 2 kb downstream of *SLC11A1* TSS, between exons 2 and 3, another DNAse I footprint was detected in several cellular backgrounds, including all those of myelo-monocytic lineage (5/27, Footprint #8, Figure 4A,B). HepG2 nuclei also displayed the footprint, which could correspond to a specific protein-DNA interaction demonstrated in HepG2 by ChIP-Seq analyses and which involves FosL2 (Figure 4C and Figure 5D). FosL2 is a leucine zipper transcription factor member of the Fos protein family that can dimerize with proteins of the JUN family (AP-1), and which is regulated by the Suppressor of cytokine signaling 3 (SOCS3) to control the expression of pro-inflammatory cytokines such as G-CSF and IL-6 in myeloid progenitor cells [177]. Interestingly, the functional *SLC11A1* polymorphism 274C/T carried by exon 3 and which affects host resistance to pediatric TB (Section 3.2) lies about 150 bp downstream this predicted regulatory element. It has been estimated that 99% of human genome lies within 1.7 kb from ENCODE-defined functional elements [150,151,152,178]. The relative proximity of *SLC11A1* polymorphism 274C/T with the predicted functional element corresponding to Footprint #8 (Figure 4A ,B) that is part of a chromatin domain apparently active in terminally differentiated CD14^+^ cells, may thus suggest that this *SLC11A1* polymorphism might influence regulation of transcription.

Two other elements potentially important for *SLC11A1* myeloid expression are located about 6 and 12 kb downstream of *SLC11A1* TSS. The first is a major footprint rather common, which is much stronger in CD14^+^ MNs than HL-60 cells (3/125, Footprint #9, Figure 4A ,B) suggesting a potential local *cis* element active in mature phagocytes. The DNAse I sensitive fragment corresponds to associations with factors such as SP1, ELF-1, p300, FOXA1, NR2A2 which were detected in HepG2 cells (Figure 5D). The second footprint (4/8, Footprint #10, Figure 4A ,B) may correspond to binding sites for various factors including RNA Pol II , TBP, EBPB, IRF1, JUND, ZBTB7A, ELF-1 which were detected in K-562 nuclear extracts. Immunoprecipitation of ELF-1 from megakaryocytic chromatin and observations of stronger footprint in NB4 compared to HL-60 cells, in the absence of reported footprint in CD14^+^ MNs, suggest that this site (Footprint #10) may contribute to developmental control of *SLC11A1* expression, similarly to predicted interactions with PU.1 and ELF-1 (Footprints #6 & 3, respectively).

To confirm the existence at *SLC11A1* locus of long range myeloid-specific *cis-*acting determinants additional developmental stages of myelopoiesis were examined using available ENCODE datasets. CD34^+^ stem cells mobilized with G-CSF are routinely prepared for repopulation of mature myeloid cells after chemotherapy for instance or for hematopoietic transplantation [179]. Accordingly G-CSF-mobilized CD34^+^ cells present a phenotype close to CMP (Figure 2A). Examination of DNAse I footprints in the chromatin of these early myeloid progenitors showed that very few candidate determinants of *SLC11A1* myeloid-specific expression were missing; essentially the region of footprints 2, 4_A__,__B_ and 5 was not DNAse I sensitive in mobilized CD34^+^ cells (Figure 4A,B). Overall the observed pattern was relatively similar to that of acute promyelocytic leukemia (APL) cells (Figure 2B), implying that activation at *SLC11A1* locus boundaries occurs early during myeloid development, in accordance with prominent roles for C/EBPβ and PU.1 [76].

Another myeloid lineage is represented by CMK cells, human acute megakaryocytic leukemia (M7) cells derived from a patient with Down syndrome. CMK cells differ from the chronic myeloid leukemia (CML) in myeloid blast crisis K-562, as the former exhibits more megakaryocytic properties and the latter erythroid markers [180]. Here again, determinants at the boundaries of the 34.6 kb locus appeared digested by DNAse I similarly to K-562 megakaryo-erythrocytic cells. However, CMK cells shared more DNAse I sensitive areas with myelo-monocytic cells implying distinct chromatin environment at *SLC11A1* locus in these two cell lines representing MEP (Figure 2 and Figure 4A,B).

In summary, combined examinations of DNAse I footprinting and ChIP-Sequencing converge to reveal different types of protein-DNA interactions. Depending on cell type(s) in which they were found these DNA-protein associations may occur at different stages of, or be maintained through, the myelo-monocytic differentiation program. Data indicate that *SLC11A1* locus spans 34.6 kb and it is activated early in myeloid development. Two candidate elements, both distal from the TSS and located at the ends of *SLC11A1* locus, nested within CTCF boundaries, are predicted to bind ETS-related factors such as PU.1 and ELF-1 as early as the CMP stage. The whole locus appears extensively sensitive to DNAse I digestion along the myelo-monocytic pathway, with increasing intensity as maturation progresses. The data support prominent roles for C/EBPβ and PU.1 in the developmental control of *SLC11A1* expression in mature phagocytes. Notably three C/EBPα/β sites, including the major TSS and two predicted upstream binding sites appear strongly mobilized to activate transcription in CD14^+^ MNs and MDMs, while another upstream site seems possibly involved earlier in promyelocytic development (NB4 cells). In contrast, most of the locus internal DNAse I hypersensitive areas disappear along the megakaryo-erythrocytic pathway implying suppression of *SLC11A1* expression in this non-phagocytic myeloid lineage.

## 6. Patterns of Histone Marks and Transcription Factor Binding at *SLC11A1* Locus

Alterations of chromatin structure, such as changes in nucleosome positioning and histone-modification patterns contribute to regulate gene transcription [181]. Using histone acetylation data to guide genomic sequence-based computational searches for TF binding sites can improve the accuracy of binding site prediction by ~40% [131]. Several histone modifications demarcate functional elements such as histone H3 lysine 4 dimethylation (H3K4me2), H3K4me3, acetylation (ac) and the presence of H2A.Z which commonly mark active promoters, and H3K4me3 marking the first nucleosome downstream the TSS. Transcribed regions are enriched for H3K36me3 and H3K79me2 while repressed genes are found in domains displaying H3K27me3 or H3K9me3, H3K9me2 marks. Regarding enhancers, these elements display enrichment for H3K4me1, H3K4me2, H3K27ac and the HAT p300, whereas elements functioning as insulators or boundary elements and structural scaffolds bind CTCF [155,182,183,184].

### 6.1. Summary of Chromatin State Segmentation

Hence myeloid specificity of *SLC11A1* transcription may be appreciated by comparing the Chromatin state segmentation results (based on ChIP-Seq data, Broad ChromHMM, for the insulator CTCF, histone marks of transcriptional activation, either methylation or acetylation, H3K4me1-3, H4K20me1, H3K36me3, H3K9ac, H3K27ac and histone methylation mark of inactive chromatin H3K27me3) between fibroblastic (NHLF), hepatocytic (HepG2), B lymphocytic (GM12878) and megakaryotic cells (data not shown).

The gene flanking *SLC11A1* in 3', *CTDSP1*, is the most active and seems expressed in all cell types considered with by decreasing order: B lymphocytes, fibroblasts, megakaryocytes and hepatocytes. Lymphocytes and fibroblasts show strong enhancer activity and highly active transcription. In contrast, *SLC11A1* is inactive in these cell types, either actively repressed by Polycomb proteins in fibroblasts or in the form of genetically inactive and tightly coiled heterochromatin in B lymphocytes (Broad ChromHMM). Though HepG2 chromatin shows weak/poised enhancer activity in the 3' part of *SLC11A1*, the 5' promoter is rather inactive. The myeloid K-562 nuclear context suggests weak level of gene transcription in the absence of enhancer or promoter activity, and not confirmed in transcriptomic analysis (ENCODE Caltech RNA-Seq, Figure 5A). Thus, as expected, *SLC11A1* chromatin context lacks histone marks of transcriptionally active gene in non myelo-monocytic lineages.

### 6.2. Histone Modifications at SLC11A1 Locus in CD14^+^ MNs

A more detailed appraisal of local histone modification landscape and that includes nuclei of CD14^+^ MNs provides crucial information to delineate areas of myelo-monocytic specific transcriptional activity and compare these with DNAse I footprinted regions (Figure 4B,D,E). Regarding *CTDSP1*, CD14^+^ MNs chromatin displays abundant marks of high transcriptional activity (H3K4me1-3, H3K36me3, H3K9ac, H3K27ac and absence of H3K27me3) that are delimited by two strong CTCF binding sites. Among other cell types, the presence of dual methylation marks of transcriptional activation/inactivation in HepG2 cells (H3K79me2/H3K27me3, respectively) may explain lower levels of *CTDSP1* transcript (Figure 5A). Concerning the putative Orf upstream of *SLC11A1*, *C2ORF62*, only HepG2 chromatin displays signs of promoter activity (H2A.Z, H3K4me1-3, H3K9ac) but no evidence of gene transcription such as *VIL* for instance (Figure 5A; and H3K79me2 and H3K36me3 marks in the body of *VIL* gene, Figure 4F). HepG2 data shows also some histone modifications at *SLC11A1*, but different from those observed in CD14^+^ MNs demonstrating strong promoter activity in several regions as well as gene transcription.

*SLC11A1* gene demonstrates in CD14^+^ MNs histone modifications in four regions of different sizes that indicate strong enhancer and promoter activities as well as gene transcription. A large 5' region starting in the middle of *C2ORF62* and spanning ~10 kb shows boundaries that seemingly correspond to DNAse I footprints which were found enriched in myelo-monocytic extracts and could indicate enhancer activity (DNAse I hypersensitive regions extending from Footprint #6 to 4_B_, Figure 4A–D). Another large region starts at *SLC11A1* TSS and extends to a common footprint found only in MNs and HL-60, and that yields stronger signal in mature cells consistent with gene expression (DNAse I hypersensitive regions #1,8,9, Figure 4A,D). The third region is smaller; it encompasses *NRAMP1* 3' UTR and exhibits marks of putative enhancer function (DNAse I hypersensitive regions #10 and 3, Figure 4A,D). The histone marks H4K20me1, H3K79me2 and H3K36me3 that typify transcribed genes link the second and third regions, implying the full coding region of *SLC11A1* is marked epigenetically. Lastly, a smaller area upstream of *SLC11A1* TSS is predominantly acetylated (DNAse I hypersensitive region #7, Figure 4A,D). All these regions show similar composition of histone marks, in which H3K27ac and H3K4me1,2 predominate thus suggesting the corresponding elements exert cell-type specific activity [154]. Indeed, in CD14^+^ MNs and MDMs, these epigenetic marks co-localize with sites of interaction with the transcription factors C/EBPβ and PU.1, both involved in human macrophage-specific gene regulation [80].

The 5' boundary of *SLC11A1* predicted upstream enhancer element is indicated by moderately intense cell-type specific histone marks H3K4me1 and H3K27ac, which correspond to the position of a DNAse I footprint detected in promyelocytic bi-potential precursors as well as protein-DNA interactions identified in megakaryotic cells and involving the factors PU.1, EGR-1 and USF1 (DNAse I Footprint #6, Figure 4A–D). The 3' boundary of this large predicted enhancer region also shows preference for cell-type specific histone marks (H3K4me1 and H3K27ac) and matches areas that were sensitive to DNAse I selectively in myelo-monocytic cells, including predicted sites for C/EBPβ and other unknown factor (DNAse I Footprints #2,4_B_, Figure 4A–D). At least four other DNAse I sensitive sequence elements were found in this region strongly marked with H3K4me1-2 and H3K9ac and H3K27ac (Figure 4A–D), which thus appears to comprise several candidate *cis* elements enhancing myelo-monocytic expression of *SLC11A1*. Several areas within this large 5' region were associated with transcription factors known to interact with macrophage specific enhancers (C/EBPβ, PU.1, EGR-2, Figure 6) [80].

The marked region that overlaps the first half of *SLC11A1* Orf is delimited in 5' by the C/EBPβ site/TSS (DNAse I Footprint #1, Figure 4A–D), which corresponds to punctual deposition of H2A.Z variant, and in 3' by a commonly recognized element (showing few marks in lymphocytes and fibroblasts, not shown) that is hypersensitive to DNAse I digestion in MNs as well as HL-60 cells (DNAse I Footprint #9, Figure 4A–D). Again, both ends of the region show rather tissue-specific composition of histone marks (H3K4me1 and H3K27ac), and the first half displays also extensive H3K9ac mark. Regarding histone methylation marks, H3K79me2 starts at *SLC11A1* TSS and covers the whole region, whereas H4K20me1 and H3K36me3 begin at the second exon or in the middle of this region and decorate the rest of the gene. This region is also notably covered with H3K4me3 mark, hereby indicating high level transcriptional activity (Figure 4A–D).

The downstream area located close to the end of *SLC11A1* Orf spans two regions hyper-sensitive to DNAse I digestion (Footprints #3 and 10), which were detected in promyelocytic bi-potential precursors (Figure 4A,B). Corresponding protein-DNA interactions identified in K-562 megakaryotic cells involve the ETS factor ELF-1. This small area is predominantly marked by H3K4me1 (Figure 6) [80], as well as H3K4me2 and H3K27ac, consistent with a putative tissue-specific enhancer role, while H3K36me3 and H4K20me1 marks indicates that transcription proceeds to the 3' end of *SLC11A1* (Figure 4A–D).

The fourth small region matches a footprint that was detected in MNs, whose position corresponds to a potential C/EBPβ site detected by ChIP-Seq in HepG2 cells upstream of the TSS (DNAse I Footprint #7, Figure 4A,B). It displays also typical histone marks of a lineage-specific transcriptional enhancer (H3K27ac, H3K4me1-2, H3K9ac, Figure 4A–D). Furthermore, in both CD14^+^ MNs and MDMs this area also displays H3K4me1 and H3K27ac marks and binds the transcription factors C/EBPβ and PU.1 (Figure 6) [80]. Hence, locus wide survey reveals excellent agreement between DNAse I footprints and both the distribution and composition of nucleosome histone marks. These data indicate that detailed analysis of candidate interacting transcription factors, including those identified by ENCODE and other ChIP-Seq analyses, will reveal the mechanistic basis of the developmental control of *SLC11A1* gene expression in myelo-monocytic cells.

In comparison, in megakaryocytic cells the upstream region is partially marked, slightly at the 5' boundary and more strongly in its middle; also the third, downstream region is well marked at the 3' end (both regions bear strong H3K4me2 and H3 ac marks suggestive of enhancer activity, Figure 4E). But interestingly, the composition of histone marks detected in K-562 cells predicts rather common *vs*. cell-type specific regulatory elements [154].

In HepG2 cells, the ends of the upstream region are marked lightly as well as the downstream 3' region (H3K4me1,2, Figure 4F) whereas the intermediate region, covering the beginning of the *SLC11A1* Orf, shows more marked boundaries (H3K4me1-3, and H3K27me3 indicative of silencing activity; Figure 4F). Hence, examining myeloid and non-myeloid cell types shows both in quantitative and qualitative aspects that the distribution of epigenetic marks indicating *SLC11A1* transcriptional activity is tissue-specific.

### 6.3. SLC11A1 Locus Predicted cis-Acting Determinants

To study further *SLC11A1* predicted regulatory regions additional sets of ChIP-Seq data were included to examine the developmental modulation of the state of histone modifications at *SLC11A1* locus (UW Histone and SYDH Histone) as well as the distribution of transcription factors potentially interacting with *SLC11A1* sequence determinants.

#### 6.3.1. 3' Distal Regulatory Element

**Figure 7:**
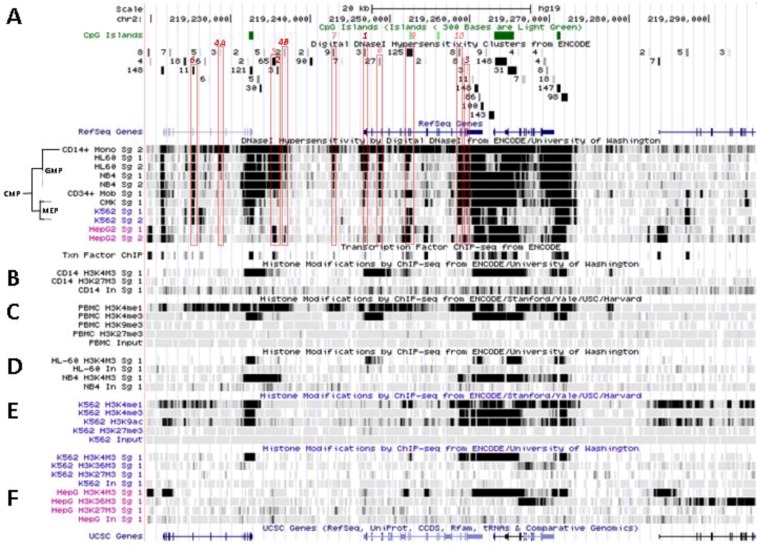
Histone modification pattern indicates *SLC11A1* transcription during myelo-monocytic differentiation. (**A**) 2q35 coordinates, CpG islands, RefSeq genes and ENCODE DNAse I hypersensitivity clusters, transcription factor ChIP-Seq, raw signals of DNAse I hypersensitivity at various stages of myelopoiesis, red boxes indicating 11 DNAse I hypersensitive sites in myelo-monocytic cell-types, as in Figure 4A,B. (**B**–**F**) ENCODE ChIP-Seq raw data for histone modifications known to correlate with gene activity (H3K4me1,3, H3K36me3, H3K9ac) or inactivity (H3K9me3, H3K27me3) found in the chromatin of CD14^+^ monocytes (**B**), peripheral blood mononuclear cells (**C**), APLs HL-60 and NB4 (**D**), CML K-562 (**E**) and hepatoma HepG2 cells (**F**).

Regarding *SLC11A1* predicted 3' regulatory element, independent data confirm that it is also marked in K-562 megakaryocytic cells (H3K4me1 and H3K4me3, Figure 4E and Figure 7E) and in promyelocytic NB4cells (H3K4me3, Figure 7D). But this H3K4me3 modification is absent from the chromatin of HL-60 cells (Figure 7D), peripheral blood mononuclear cells (PBMCs, only H3K4me1, Figure 7C) and apparently CD14^+^ MNs (Figure 4D and Figure 7B). In MNs and MDMs, the mark H3K4me1 is the most intense (Figure 4D and Figure 6B), which together with the presence of H3K27ac is consistent with cell-type specific enhancing activity.

The data suggest that histone modifications observed in K-562 megakaryocytic cells (H3K4me1-3; H3K9ac and H3K27ac) and in NB4 promyelocytic cells (H3K4me3), as well as protein interactions revealed by ChIP-Seq analyses at relevant DNA sites and involving RNA Pol II, c-Myc and Max (Figure 3H), together indicate that the functional state for this 3' predicted regulatory element is distinct from the specific activity acquired along the myelo-monocytic differentiation pathway.

Interestingly, another myeloid pathway, erythroid differentiation, stimulates the expression of transferrin receptor 1 (TfR1) and ferrochelatase (Fech) and increases iron uptake and heme biosynthesis as a result of more c-Myc-Max functional complexes interacting with TfR1 and Fech promoter elements [185]. Because *SLC11A1* may also increase phagocyte cytoplasmic Fe content it would seem plausible that c-Myc-Max functional complexes have a positive impact on *SLC11A1* expression, albeit early in myeloid differentiation. Indeed, significant interactions were detected between c-Myc-Max complexes, RNA Pol II and *SLC11A1* locus in promyelocytic NB4 cells (Figure 3G). In HL-60 cells RNA Pol II did not appear bound to this element or marginally (Figure 3F). These data further support that HL-60 chromatin resembles more that of CD14^+^ MNs and MDMs than NB4 promyelocytic cells.

In another set of data, weak association of C/EBPβ with one of *SLC11A1* 3' distal elements was found in the chromatin of CD14^+^ MNs and MDMs (Footprint #10, Figure 6A,B) [80]. EGR-2 binding was also detected in MDMs at an adjacent site as well as another downstream site corresponding to *SLC11A1* locus 3' insulator. Both EGR-1, found to associate at *SLC11A1* locus 5' boundary (Section 5.2.2), and EGR-2 were involved in the induction of myelomonocytic differentiation of U937 and HL-60 leukemia cells as well as in MN activation [186].

Thus *SLC11A1* 3' end comprises predicted regulatory elements that may be active early in myeloid development, including NB4 and K-562 cells, and exerting regulatory activity through the action of transcription factors such as ELF-1, C/EBPβ , EGR-2, c-Myc and Max together with RNA Pol II. In addition, this element presents a cell-type specific profile of histone modifications that is shared by CD14^+^ MNs and MDMs.

#### 6.3.2. 5' Distal Regulatory Element

The predicted *SLC11A1* 5' enhancer is organized differently from the 3' regulatory element as it spreads bi-directionally from a rather common core element that is found in other hematopoietic lineages, myeloid (K-562) or not (B lymphocytes), and to a lesser extent in lung fibroblasts, but absent from HepG2 cells (Figure 4D–F).

In MNs, histone modifications extend towards both ends of a large regulatory domain. In the 5' half and at the 3' end, H3K4me1,2 and H3K27ac marks dominate suggesting myelo-monocytic specificity whereas most of the 3' half, which is marked also with H3K4me3 and H3K9ac, rather indicates more common regulatory elements (Figure 4D and Figure 7B). Accordingly, a strong and spread H3K4me1 signal is also detected together with more restricted H3K4me3 mark in PBMCs, which include MNs (Figure 7C). But in ontogenically divergent myeloid cells (K-562), this H3K4me1 mark is detected only over the 5' arm while other marks are limited to the common core element (Figure 4E and Figure 7E). In promyelocytic intermediates, the chromatin of NB4 cells displays H3K4me3 pattern similar to MNs, whereas PBMCs and HL-60 cells show marks more confined to the core segment and resembling the pattern found in megakaryocytic K-562 cells (Figure 7D,E).

In addition, ChIP-Seq analyses of RNA Pol II distribution demonstrate several interactions with relevant DNA segments in HL-60 cells (Figure 3F). Interestingly, in NB4 cells interactions with RNA Pol II are restricted to the core element although the other areas display evidence for the presence of c-Myc-Max complexes (Figure 3G), which correspond to interactions with PU.1 in HL-60 cells (Figure 3F). Indeed, PU.1 binding is distributed on both arms of the 5' enhancer in MNs and MDMs and co-detection of PU.1 and EGR-2 at the 5' boundary of the enhancer domain (Footprint #6, Figure 6) supports a role for the corresponding site in myelomonocytic differentiation [143]. In contrast less abundant interactions are observed at fewer sites between *SLC11A1* predicted 5' enhancer and c-Myc Max complexes and RNA Pol II in K-562 cells implying reduced activity compared to CD14^+^ MNs (Figure 3H).

Current data on *SLC11A1* predicted 5' and 3' distal regulatory elements thus provide different pictures but support similar priming early in myeloid development, such as the CMP stage that is a precursor of both megakaryo-erythrocytic and myelo-monocytic lineages (Figure 2). These 5' and 3' distal elements may become poised for enhancing under the impulse of c-Myc-Max complexes or PU.1 factor for instance, at a stage close to promyelocytic precursors. The large 5' element presents a profile fit for enhancer function that becomes activated late in the myelo-monocytic differentiation program in accordance with *SLC11A1* mRNA accumulation data [70]. In contrast, the 3' element may exert dual function, including in myeloid cells that do not express *SLC11A1*.

Compared to NB4 cells HL-60 chromatin state shows differences which could be due to heterogeneity among APLs. Differential pharmacological modulation of histone modifications have previously been reported with NB4 and HL-60 cell lines; such as H4 acetylation in the G-CSF receptor promoter at the C/EBPα binding site in HL-60 but not in NB4 cells [187]. Interactions detected in HL-60 cells between *SLC11A1* putative 5' enhancer and RNA Pol II, GABP and PU.1 (Figure 3F) indicate the possibility that the relevant elements are active in these cells whereas they are not in NB4 and K-562 cells. It will be useful to examine marks such as H3K4me1 and H3K4me2 as well as H3K9ac, H3K27ac and H4ac to support the cell-specificity and activation stage of *SLC11A1* predicted 5' enhancer domain in APL cells.

The data raise the possibility that *SLC11A1* upstream enhancer may be partly activated in HL-60 cells with relatively little activity in its 3' part. The intensity of DNAse I footprinting appeared weaker in this area notably the region corresponding to the C/EBPβ/STAT3/ C/EBPβ/MAFK putative regulatory hub. Based on cell-type specific histone modifications marks observed in CD14^+^ MNs it seems very likely that this region is mobilized in late stages of myelo-monocytic differentiation, such as during VitD-induced monocytic differentiation of HL-60 cells. Other sets of data support this proposition, showing several sites of interaction with PU.1, from the 5' boundary of the enhancer domain to the TSS both in CD14^+^ MNs and MDMs (Figure 6). In addition, C/EBPβ apparently binds to the predicted site 5' adjacent to the STAT3 responding element, co-localizing with PU.1, both in CD14^+^ MNs and MDMs (Footprint #5, Figure 6). Interestingly, PU.1 binding in the area differs between MNs and MDMs, with additional binding in 3' for the latter suggesting potential regulatory role in late stages of myelo-monocytic differentiation.

In contrast, the central core element of this large 5' enhancer corresponds to a common DNAse I footprint (121 cell types) which matches DNA fragments pulled by ChIP-Seq that targeted a variety of transcription factors, including RNA Pol II, c-MYC-MAX, EGR-1, ETS-1 and ELF-1. These factors are similar to those potentially binding at the 5' boundary of this large enhancer element, as well as at *SLC11A1* distal 3' regulatory element where they correspond to more specific DNAse I footprints found in only 11 and 8 cell types, including K-562 cells (Footprints #6 and #10, respectively, Figure 4A,B). This prominent and frequently found (121 cell types) DNAse I footprint matches also a CpG island (Figure 3A), which supports a functional regulatory role [136]. In addition, it also corresponds to an area delimited by endonuclease accessible sites (NAS) in the chromatin of the CD34^−^ monocytic cells, but not in their CD34^+^ HSCP (EIO-JCVI NAS, Figure 5B) [76,156], thereby confirming activation of this element during myeloid differentiation.

#### 6.3.3. Transcription Start Site and Gene Body

In MNs, histone modifications around *SLC11A1* proximal promoter cover about half of the coding gene. Starting at the TSS this area extends to a common DNAse I footprint which gave a strong signal in MNs that corresponds in ChIP-Seq analysis of HepG2 cells to DNA associations with transcription factors such as SP1, ELF-1, p300, FOXA1, NR2A2 (Footprint #9, Figure 7A). This broad stretch, which overlaps a small CpG island, also harbours extensive H3K4me1-3, H3K79me2 and H4K20me1 marks; it is highly acetylated in its first half and shows H3K36me3 mark in the second part, which co-distributes with H4K20me1 along the body of *SLC11A1* (Figure 4D and Figure 7B) and together indicate transcriptional activity. This is confirmed by detection of strong binding of both PU.1 and C/EBPβ at the TSS in CD14^+^ MNs and MDMs [80] (Figure 6B, Footprint #1) which suggest that PU.1 and C/EBPβ cooperate to activate *SLC11A1* transcription.

The H3K4me3 mark indicative of transcriptional activity shows conserved pattern in PBMCs (Figure 7C) and in HL-60 promyelocytes albeit less abundant (Figure 7D). Yet H3K4me3 was not detected in the TSS area neither in promyelocytic NB4 nor in megakaryocytic K-562 cells (Figure 7D,E), supporting that the promoter is only activated late in the myelo-monocytic differentiation program. Such presumed difference in activity is supported by the absence in NB4 and K-562 chromatin of RNA Pol II at *SLC11A1* TSS, and in several other places of the predicted 5' enhancer (Figure 3F–H), as well as by reduced DNAse I footprinting in the promoter area (in NB4 compared to HL-60) and in the 5' enhancer area (K-562; Figure 7A).

#### 6.3.4. Distal Promoter

The last element identified represents a distal promoter constituent that likely interacts with C/EBPβ , and preferentially in mature myelo-monocytic cells (Footprint #7, Figure 7A). In CD14^+^ MNs the area shows the cell-type specific marks H3K27ac and H3K4me1 (Figure 4D) and it corresponds to a prominent DNAse I footprint which matches a DNA segment interacting with C/EBPβ in HepG2 cells (Figure 4C). The H3K4me1 methylation mark was found as well in the chromatin of PBMCs (Figure 7C), but was not assayed in HL-60 or NB4 promyelocytic cells. Arguably, *SLC11A1* distal promoter element becomes fully activated in the final stages of monocytic differentiation, in accordance with the established role of C/EBPβ mediating VitD-induced expression of *SLC11A1* and with cell-type specific histone marks observed in CD14^+^ cells. This interpretation is supported by strong association of C/EBPβ in CD14^+^ MNs and MDMs (Figure 6, Footprint #7) [80].

Comparing NAS-Seq data from CD34^−^ monocytic cells and their CD34^+^ HSC progenitors (EIO-JCVI NAS) [76,156] allows confirming this proposition. Monocytic cells show nuclease sensitivity for both the predicted distal promoter site and *SLC11A1* TSS (Figure 5B), which can equally interact with C/EBPβ (Figure 6). In contrast, these sites display no sensitivity to nuclease in self-renewing CD34^+^ HSCs (Figure 5B), which are not committed to any hematopoietic pathway (Figure 2A), supporting that *SLC11A1* expression is specifically controlled during myelo-monocytic development. 

Lastly, the chromatin state of non-hematopoietic hepatocellular carcinoma HepG2 cells demonstrates low level transcriptional activity (ENCODE Caltech RNA-Seq, Figure 5A) which correlates with histone marks at the promoter level (Figure 4F): though H3K4me1-3 marks are present at *SLC11A1* TSS the relative proportion of H3K4me3 is low; H3 acetylation is present only downstream of the TSS, whereas the H3K27me3 inhibitory mark is found starting upstream of the TSS [155,188]. These marks suggest transcription might be possible, and potentially supported through some activity at the 5' and 3' regulatory elements as well; but little if any RNA Pol II found interacting at *SLC11A1* TSS (Figure 3I) indicates that HepG2 chromatin prevents transcription. The downstream neighbour genes *CTDSP1* and *VIL* are transcriptionally active in HepG2 cells but separated from *SLC11A1* by one or two CTCF insulator sites, respectively. Hence, it is unclear at present why a myelomonocytic-specific gene is relatively accessible, but apparently inactive, within the chromatin of hepatoma cells.

## 7. Hypothesis on the Developmental Control of *NRAMP1* Expression in Myeloid Cells

Combined chromatin signatures and association with RNA Pol II are highly correlated with enhancer activity [189,190]. The data from ChIP-Seq analyses of RNA Pol II interactions may thus indicate activity of regulatory determinants. The 3' element located at the end of *SLC11A1* demonstrates the presence of RNA Pol II in the megakaryocytic and promyelocytic bi-potential cells K-562 and NB4, respectively. Apparently less RNA Pol II accumulated in this area in the other promyelocytic cell line HL-60, which however binds RNA Pol II notably at *SLC11A1* TSS. This suggests that activity of this 3' distal element may differ between *NRAMP1* expressing and non-expressing myeloid cells. 

In addition, K-562 and NB4 cells lack RNA Pol II association at sites corresponding to DNAse I hypersensitive elements in the predicted upstream enhancer element. The more 5' site is found mainly in myeloid cells (11) and the other (65) is more common (Footprints #6 and #5, respectively). The presence at these two sites of c-Myc-Max complexes in NB4 and not K-562 cells may thus be interpreted as myelomonocytic-specific recruitment of a transcription factor, which is one of the first steps of enhancer nucleosome remodeling [106,190]. This interpretation is consistent with data from DNAse I digestion studies, which show that both K-562 and CMK myeloid cells lack several footprints in this area and by this criterion appear less active than cells representing the CMP stage (CD34^+^ mobilized, Figure 2 and Figure 7B).

It is thus possible to propose a hypothetical progression of events leading to the activation of *SLC11A1* transcription: Chromatin opening of *SLC11A1/NRAMP1* gene is proposed to start at the locus boundaries. The 3' regulatory element, which is adjacent to a CTCF-specific element that insulates from nearby *CTDSP1* gene, may bind “pioneer” transcription factors at an early myeloid stage before divergence of the megakaryo-erythrocytic and myelo-monocytic lineages (CMP stage). The co-existence of DNAse I footprints matching site-specific transcription factor interactions in an area of specific histone methylation and acetylation marks, together predict some activity of this element in megakaryocytic cells. Site-specific associations with transcription factors (c-Myc-Max, RNA Pol II) imply that this 3' regulatory element is also active in NB4, and hence not sufficient to trigger *SLC11A1* transcription in committed myeloid progenitors. Histone modification profile in CD14^+^ MNs predicts cell-type specific activity of *SLC11A1* 3' regulatory element, supported by site-specific associations reported with C/EBPβ and EGR-2 [80]. It is possible to reconcile these findings by postulating that this 3' element exerts dual activity, either enhancer or repressor for instance, depending on the myeloid developmental stage and transcription (co-)factor(s) recruited.

Similarly, the core of *SLC11A1* 5' enhancer and its 5' boundary are also mobilized early during myeloid differentiation. However, it is only along the myelo-monocytic lineage that *SLC11A1* 5' enhancer further recruits transcription factors and becomes activated, as seen in DNAse I footprints and histone modification marks from NB4 and HL-60 promyelocytic cells and in CD14^+^ MNs. Based on site-specific associations with several transcription factors (PU.1, EGR-2, C/EBPβ , c-Myc-Max and RNA Pol II) it is suggested that this upstream enhancer is targeted by multiple sequence-specific transcription factors and becomes fully activated in CD14^+^ MNs and MDMs, and at least partly in promyelocytic HL-60 cells which demonstrate bound RNA Pol II at *SLC11A1* TSS. Consequently, defective activation of *SLC11A1* 5' myelo-monocytic enhancer (lack of RNA Pol II and/or PU.1 and C/EBPβ association with select sites) may contribute to explain the absence of transcriptional activation at *SLC11A1* promoter in NB4 and K-562 cells, which represent progenitors committed towards the myelo-monocytic or megakaryo-erythrocytic pathway, respectively.

One DNAse I footprint within the 5' arm of the upstream enhancer (#4_A_) and four others distributed over *SLC11A1* promoter and first half of the gene body (#7,1,8,9) are less intense in NB4 cells compared to HL-60 and CD14^+^ MNs. The locations of these footprints correspond in CD14^+^ MNs to areas where H3 histones are modified by acetylation and exhibit K4me1,2 marks typical of cell-type specific enhancer elements [154,155,190], and these areas correspond to binding sites for PU.1 and/or C/EBPβ in CD14^+^ MNs and MDMs. Hence defective activation of myelomonocytic-specific upstream enhancer elements such as Footprint #4_A_ (Figure 6) constitutes a likely explanation for the lack of *SLC11A1* transcriptional activation observed in NB4 promyelocytic cells.

Lack of enhancer activation may be the result of repressor activity, which may compete with activator proteins for shared DNA binding sites, inhibit or quench adjacent activators bound to a common regulatory element, or repress directly RNA Pol II transcription complex [191,192]. Regarding the DNAse I footprint #10 that is detected in *SLC11A1* 3' regulatory element with similar intensities in NB4 and K-562, representing twofold the signal in HL-60 cells, its functional association with RNA Pol II in cell types not expressing *SLC11A1*, *i.e.*, NB4 and K-562, suggests potential repressor or incomplete enhancer activity. Similarly, association in K-562 and NB4 cells of RNA Pol II with a footprint that overlaps the CpG island forming the core of *SLC11A1* large 5' enhancer suggests insufficient activator role, unable to stimulate *SLC11A1* transcription in these cells. Indeed, strong association of RNA Pol II with a nearby 3' site in NB4 cells and not in HL-60 cells supports the possibility that regulatory sites may be found as well at the core the large 5' enhancer element. NB4 cells lack several associations of RNA Pol II with sites at both ends of the 5' enhancer area indicating that this region acquires capacity to activate *NRAMP1* transcription in mature myelo-monocytic cells such as MNs and HL-60 differentiated cells.

## 8. Conclusion

*SLC11A1/NRAMP1* is specifically expressed in professional phagocytes and encodes the archetype eukaryotic cell defense against shortage of metal ions such as Fe and Mn. SLC11 membrane carriers catalyze proton-dependent divalent metal import into the cytoplasm and NRAMP1 protein acts intracellularly in the membrane of phagosomes containing either material for recycling or potential invading microbes. NRAMP2/DMT1 is more ubiquitously expressed at the cell surface and in early endosomal network; it is essential for cell and organism metal absorption. NRAMP1 and 2 parologs are present in Sarcopterygii whereas ray-finned fishes possess only Nramp2 homolog(s) mediating both functions. All Sarcopterygii including land vertebrates deposit Fe in dental enamel, a process aided by Nramp2/Dmt1 and Fpn1 in mouse ameloblasts. Due to the reaction of Fenton SLC11 activities may impact the production of reactive oxygen species so that their expression also has to be co-ordinately regulated with metal storage and/or export functions as well as redox sensitive, cyto-protective transcriptional responses.

*SLC11A1/NRAMP1* tissue specific expression is controlled primarily at the transcriptional level and specific sequence determinants in the promoter region have been defined functionally using the myelo-monocytic model cell line HL-60. Monocytic differentiation induced with VitD yields CD14^+^ cells with functional properties reflecting steady-state anti-inflammatory conditions; they express *SLC11A1* under the control of C/EBPβ which binds the major TSS. It is likely that PU.1 is also required to activate *SLC11A1* transcription. Macrophage-like differentiation induced with PMA, or activation of MN-like cells with bacterial LPS both stimulate pro-inflammatory functions and *SLC11A1* expression; binding of AP-1-like ATF3/JunB dimers allows to recruit the SWI/SNF chromatin remodeling complex that facilitates binding of HIF-1α which upregulates *SLC11A1* transcription.

Genome-wide analyses of chromatin status and transcription factor binding in various tissues have revealed that 80% of the genome exert functional role [151,152,178]. ENCODE [150,151] high throughput large scale coordinated analyses thus enable to survey potential functional sites at any locus and usefully complement targeted studies aimed at characterizing the roles of *cis*-elements and *trans*-acting factors involved at different steps controlling the developmental regulation of gene expression.

Examining these and other data regarding *SLC11A1* allowed to confirm the major TSS indicated by the presence of RNA Pol II in HL-60 myelo-monocytic cells, support the prominent role of C/EBPβ and indicate an important role for PU.1, not only at the TSS but also upstream enhancer elements. The data reveal two distal regulatory elements located close to *SLC11A1* locus boundaries and apparently mobilized early during the myeloid developmental program, and allow to predict several regulatory sites within the large 5' enhancer or scattered along the body of *SLC11A1* gene. The regulation of *SLC11A1* expression is thus significantly more complex than initially anticipated.

Data from cell types representing discrete steps in the myelopoietic pathway also suggest a plausible sequence of events controlling gene expression. *SLC11A1* locus is inactive in HSCs but displays activation marks at the CMP stage. Whereas *SLC11A1* locus activation recedes in the megakaryo-erythrocytic pathway, the gene acquires along the myelo-monocytic lineage histone modification marks and displays site-specific associations with transcription factors, including RNA Pol II, which together indicate tissue-specific activation of transcriptional activity.

Studying leukemia cells representing committed myeloid progenitors with bi-potential differentiation capacity not only enables longitudinal study of the control of gene expression but to also address differences in locus activation, which may result from genetic and/or epigenetic variations such as those observed at *SLC11A1* locus between HL-60 and NB4 cells or K-562 and CMK cells, and which in turn may inform on (epi)genetic processes relating to leukemogenesis.

Current data confirm that HL-60 cells provide a model representative of human myelo-monocytic differentiation. However, in the other promyelocytic cell line NB4, *SLC11A1* is not prone to transcriptional activation. Epigenetic differences between HL-60 and NB4 cells alter their response to VitD, which induces differentiation by genomic (VDR) or non-genomic mechanisms, respectively, [95,187,193]. Studying *SLC11A1* locus can thus help evidence molecular aberrations that occur in myelo-monocytic leukemia.

*SLC11A1* lies within a 34.6 kb insulated locus that comprises an abundance of predicted *cis*-elements which may exert positive or negative effects on the activity of *SLC11A1* proximal promoter and gene expression, not only during myeloid development but also due to phenotypic plasticity of mature cells reaching blood and/or tissues and depending on the immune context, being steady-state or inflammatory conditions. Chromatin remodeling is key to macrophage polarization as the Jumonji-D (JmjD) domain Jmjd3 protein catalyzing H3K27 demethylation to revert gene silencing is essential for M2 macrophage polarization, whereas M1 activation leads to H3K4 trimethylation on cytokine gene promoters, implying active gene transcription [194].

*SLC11A1* basal expression in VitD polarized/CD14^+^ (M2) cells depends on C/EBPβ and correlates with H3K4me3 modification in the absence of H3K27me3 mark. Experimental validation of the developmental control of *SLC11A1* expression will provide a useful framework to further analyze how macrophage polarization and response to infectious and/or inflammatory stimuli modulate *SLC11A1* transcription. In addition, SLC11A1/NRAMP1 function represents significant pro-inflammatory potential as the protein catalyzes cytoplasmic accumulation of labile iron. To avoid tissue damage such potential pro-inflammatory activity must be tightly regulated in coordination with immune status as well as cytoprotective responses.

Hence M2 macrophages geared to recycle apoptotic cells and iron, fuel mitochondrial biogenesis and oxidative metabolism but limit the production of reactive oxygen species (ROS). ROS production resulting from mitochondrial respiration is controlled by modulating the expression of uncoupling proteins, e.g., UCP2 [195], which promote fatty acid metabolism and regulate mitochondrial membrane potential through long chain fatty acid anion/H^+^ symport [196]. Continuous phagocytosis of apoptotic cells depends on UCP2 expression whereas ingestion of synthetic and infectious particles is not affected by UCP2 [197,198]. In contrast, macrophage M1 activation down-regulates UCP2 and increases ROS-dependent MAPK pro-inflammatory signaling [195]. Up-regulation by HIF-1α of *NRAMP1* transcription induced by bacterial LPS or the pharmacological agent PMA thus may contribute to M1 macrophage pro-inflammatory activity.

Understanding the regulation of *SLC11A1* locus activity in myelo-monocytic cells is expected to further gain in precision and comprehensiveness thanks to continuous large scale efforts aiming to depict all the functional elements that may affect gene expression. Data analyses combining different myeloid cell types provide a road map for future targeted studies aimed at elucidating the mechanisms that govern *SLC11A1* transcriptional activation and its regulation by demonstrating the relevant steps experimentally.

Functional validation of *SLC11A1* SNPs most consistently linked to resistance/susceptibility to TB provides another perspective on modulations of *SLC11A1* expression. The proximal promoter comprises a polymorphic (TG)_n_ repeat that contains two sites for the transcription factor HIF-1α which typifies M1, pro-inflammatory macrophages. Activation with LPS, or macrophage-like differentiation of promyelocytes, induce binding of an AP-1 related transcription factor complex (ATF3/JunB) immediately upstream of the (TG)_n_ repeat. The ATF3/JunB complex recruits components of the SWI/SNF chromatin remodeling complex which actively convert the (TG)_n_ repeat conformation into Z-DNA that allows binding of the transcriptional activator HIF-1α. Alleles different from allele 3, which are less efficient at recruiting HIF-1α have been associated with susceptibility to TB.

*SLC11A1* predicted 3' distal regulatory element also comprises polymorphisms that were linked to TB resistance/susceptibility such as the mutation D543N. The corresponding amino acid replacement is located in a region of the protein lacking sequence conservation and it is not established whether it affects protein activity. But *SLC11A1* coding exon (XV) spans a DNA segment hypersensitive to DNAse I digestion in myelo-monocytic cells which was functionally associated with the transcription factors ELF-1, EGR-2 and C/EBPβ. It seems thus possible that *SLC11A1/NRAMP1* mutation D543N affects gene expression rather than protein activity. This suggestion is supported by occurrence of a nearby 3' UTR polymorphism that was linked to increased TB resistance in urbanized populations.

Identifying all the SNPs that are located in areas of open chromatin such DNAse I hypersensitive sites which may influence *SLC11A1* gene regulation during myeloid development and/or activation of mature cells is ultimately desirable. These SNPs may perturb transcription factor binding and activity or impede allelic nucleosome marking and remodeling and thus impact gene expression [199]. The identification of variants associated with susceptibility/resistance to infectious diseases such as TB will be useful to further probe the relationship between (mainly) non-coding polymorphism at *SLC11A1* locus and pathogenesis of infectious and/or immune diseases.
